# Curcumin-driven reprogramming of the gut microbiota and metabolome ameliorates motor deficits and neuroinflammation in a mouse model of Parkinson’s disease

**DOI:** 10.3389/fcimb.2022.887407

**Published:** 2022-08-10

**Authors:** Can Cui, Yingying Han, Hongxia Li, Hongxiang Yu, Bei Zhang, Gang Li

**Affiliations:** Department of Neurology, Shanghai East Hospital, School of Medicine, Tongji University, Shanghai, China

**Keywords:** Parkinson’s disease, curcumin, gut microbiota, tyrosine, dopa

## Abstract

**Background:**

Parkinson’s disease (PD) is a common neurodegenerative disorder, accompanied by motor deficits as well as gastrointestinal dysfunctions. Recent studies have proved that the disturbance of gut microbiota and metabolism contributes to the pathogenesis of PD; however, the mechanisms underlying these effects have yet to be elucidated. Curcumin (CUR) has been reported to provide neuroprotective effects on neurological disorders and modulate the gut flora in intestinal-related diseases. Therefore, it is of significant interest to investigate whether CUR could exert a protective effect on PD and whether the effect of CUR is dependent on the intestinal flora and subsequent changes in metabolites.

**Methods:**

In this study, we investigated the neuroprotective effects of CUR on a mouse model of PD induced by 1-methyl-4-phenyl-1,2,3,6-tetrahydropyridine (MPTP). 16S rRNA sequencing was performed to explore the profile of the gut microbiota among controls, MPTP-treated mice and CUR-treated mice. Then, antibiotic treatment (ABX) and fecal microbiota transplantation (FMT) experiments were conducted to examine the role of intestinal microbes on the protective effects of CUR in PD mice. Furthermore, ultra-high-performance liquid chromatography-mass spectrometry (UHPLC-MS)-based metabolomics analysis was used to identify the landscape of the CUR-driven serum metabolome. Finally, Pearson’s analysis was conducted to investigate correlations between the gut flora-metabolite axis and CUR-driven neuroprotection in PD.

**Results:**

Our results showed that CUR intervention effectively improved motor deficits, glial cell activation, and the aggregation of α-synuclein (α-syn) in MPTP-treated mice. 16S rRNA sequencing showed elevated abundances of *Muribaculaceae*, *Lactobacillaceae*, *Lachnospiraceae* and *Eggerthellaceae* but depleted abundances of *Aerococcaceae* and *Staphylococcaceae* in CUR-treated mice when compared with MPTP mice. ABX and FMT experiments further confirmed that the gut microbiota was required for CUR-induced protection in PD mice. Serum metabolomics analysis showed that CUR notably upregulated the levels of tyrosine, methionine, sarcosine and creatine. Importantly, strong correlations were identified among crucial taxa (*Aerococcaceae*, *Staphylococcaceae*, *Muribaculaceae*, *Lactobacillaceae*, *Lachnospiraceae* and *Eggerthellaceae*), pivotal metabolites (tyrosine, methionine, sarcosine and creatine) and the motor function and pathological results of mice. CUR treatment led to a rapid increase in the brain levels of tyrosine and levodopa (dopa) these changes were related to the abundances of *Lactobacillaceae* and *Aerococcaceae*.

**Conclusions:**

CUR exerts a protective effect on the progression of PD by modulating the gut microbiota-metabolite axis. *Lactobacillaceae* and *Aerococcaceae*, along with key metabolites such as tyrosine and dopa play a dominant role in CUR-associated neuroprotection in PD mice. Our findings offer unique insights into the pathogenesis and potential treatment of PD.

## 1 Introduction

Parkinson’s disease (PD) is the second most common progressive neurodegenerative disorder and has a number of clinical manifestations, including motor deficits, resting tremor, rigidity, bradykinesia and gait impairments (Nalls et al., 2014; Heinzel et al., 2019). The loss of dopaminergic (DA) neurons in the substantia nigra pars compacta (SNpc)-striatum axis and α-synuclein (α-syn) aggregation in the Lewy bodies are recognized as the typical pathological features of PD (Dickson et al., 2009). At present, the pathogenesis of PD remains ambiguous and the therapeutic strategies for PD are only classical methods that can only ameliorate the motor symptoms but are ineffective in slowing the progression of disease (Armstrong and Okun, 2020). Recently, neurologists have observed that gastrointestinal (GI) problems, such as constipation, begin decades before motor dysfunction in PD patients (Bloem et al., 2021). It has also been shown that the accumulation of misfolded α-syn protein begins in the enteric nervous system decades before the presence of symptoms in the central nervous system (CNS) (Schapira et al., 2017). Moreover, several studies have supported the fact that PD might originate from the GI tract, thus suggesting the crucial role of the intestine in PD progression (Abbott et al., 2001; Tan et al., 2014; Fasano et al., 2015).

The human gut microbiota, colonized within the GI tract, is essential to communication between the gut and the host (Ley et al., 2006; Armstrong and Okun, 2020). It was previously found that microbes play a vital role in the pathogenesis of PD *via* the modulation of metabolites, the integrity of the GI epithelial barrier and immune function (Jiang et al., 2017). Furthermore, an imbalance in the composition and function of gut bacteria is thought to be associated with PD pathology. PD patients who exhibited GI symptoms were previously shown to exhibit a distinct composition and function of intestinal microbiota when compared with healthy controls (Hill-Burns et al., 2017). Furthermore, the gut flora from PD patients exacerbated motor deficits when transplanted into germ-free mice, whereas this effect is reversed following antibiotic treatment, thus emphasizing a causal role for gut microbes in the pathology of PD (Sampson et al., 2016). As a classical mechanism of host-microbe crosstalk, gut microbes are reported to produce a range of metabolites, such as amino acids and secondary bile acids, that then accumulate in the peripheral serum (Sanna et al., 2019; Liu Y et al., 2020). Notably, the gut microbiota can regulate the metabolism of aromatic amino acid (AAA) which may regulate immune function, along with metabolic and neuronal responses in the CNS (Liu Y et al., 2020). Tyrosine is a vital AAA that is mainly synthesized from phenylalanine, the precursor to levodopa (dopa) that is commonly used to treat PD (Bressman and Saunders-Pullman, 2019). However, the specific relationship between aberrant gut flora and the pathogenesis of PD has yet to be elucidated.

Curcumin (CUR) a low molecular weight polyphenolic compound obtained from *Curcuma longa* is widely employed in traditional Chinese medicine (Aggarwal and Harikumar, 2009). Emerging data suggest that CUR benefits the host *via* a number of mechanisms, including anti-inflammatory, antioxidant, and anti-cancer properties (Liu FH et al., 2016; Kocyigit and Guler, 2017; Jaiswal et al., 2017). Furthermore, CUR was shown to contribute to PD treatment through multiple pathways, including the prevention of reactive oxygen species (ROS) production, glial cell activation, α-syn aggregation and neuronal cell apoptosis (Chen et al., 2006; Lee et al., 2007; Jiang et al., 2013; Peng et al., 2017).

However, poor bioavailability and the lack of permeability in the blood-brain barrier have led to widespread speculation as how CUR could exert such effects in PD (Wahlang et al., 2011). We hypothesized that the peripheral influences of CUR might be more important than direct influences on the CNS. Indeed, CUR is known to restore microbial dysbiosis and can improve gut barrier function, reduce inflammation and modulate circulating metabolites, thus playing a protective role in many diseases (Huang et al., 2021; Islam et al., 2021). However, whether CUR could have a protective effect on PD *via* gut microbiota remains unknown.

In this study, we created a mouse model of PD with 1-methyl-4-phenyl-1,2,3,6-tetrahydropyridine (MPTP) and demonstrated that CUR improved motor impairments and reduced DA neuronal loss in PD mice. We also identified compositional shifts in the gut flora and histological changes in the colon in response to CUR administration. The neuroprotective effect of CUR was blocked by antibiotic treatment (ABX) and reversed by fecal microbiota transplantation (FMT) experiment. Metabolomics analysis further revealed that the serum levels of tyrosine, cysteine, methionine, sarcosine, and creatine were higher in the CUR group when compared with the MPTP group. Correlation analysis identified one potential pathway involving microbiota-associated effects on circulating amino acid metabolism. CUR intervention also increased the levels of tyrosine and dopa in the brain of PD mice. These findings suggest that the neuroprotective effects of CUR in PD involve CUR-induced changes in the gut bacteria and alterations in the levels of key amino acids.

## 2 Methods

### 2.1 Animals and treatments

Male C57BL/6 mice (age 6 to 8 weeks, weighing 20−30g), were purchased from Shanghai SLAC Laboratory Animal Co., Ltd and were housed under a specific pathogen-free environment (23 ± 2°C, 45 ± 5% humidity, 12/12 h light/dark). The mouse procedures in this study were approved by the ethics committee of Tongji University. All experiments were conducted in adherence to the National Institutes of Health Guidelines for the Care and Use of Laboratory Animals (No. 55 issued by the Ministry of Health, revised 1998).

Reagents and drugs were prepared immediately before their use. MPTP-induced PD mice model was established as described previously (Liu H et al., 2020). In brief, the solution containing 30 mg/kg MPTP (Cat# M0896, Sigma-Aldrich, St Louis, United States) was prepared using sterile saline and the mice were intraperitoneally injected for 5 consecutive days to construct the sub-acute PD model, whereas control mice were received saline. For the CUR intervention experiment, CUR (25,100,400 mg/kg) (Cat# C1386, Sigma-Aldrich) was prepared in sterile saline and given through intragastric administration daily for 4 weeks starting 2 weeks before MPTP injection. The dose selection of curcumin is based on previous relevant studies (Szymusiak et al., 2016; Muthian et al., 2018; Abdul-Latif et al., 2021).

In the antibiotic treatment experiment, the antibiotic solution was freshly prepared with 1 g/L ampicillin (Cat# A100339, Sangon Biotech, Shanghai, China), 1 g/L neomycin (Cat# A6610366, Sangon Biotech), 1 g/L metronidazole (Cat# A600633, Sangon Biotech), and 0.5 g/L vancomycin hydrochloride (Cat# A600983, Sangon Biotech) in sterilized water for immediate use similarly to previous studies (Sampson et al., 2016). The antibiotic administration was performed for 5 weeks to remove approximately 99% of intestinal flora.

In the fecal microbiota transplantation experiment, fresh fecal pellets were collected from MPTP treated mice, controls, and CUR treated mice, and then diluted in PBS (1 fecal pellet/ml), and steeped for 15 min. The stool was centrifuged at 1000 rpm for 5 min at 4°C. Then, the suspension was centrifuged at 8000 rpm and 4°C for 5 min to obtain the total bacteria. The bacteria were filtered twice in PBS and mixed with glycerol at a final concentration of 20% and stored at -80°C until transplantation. Each recipient mouse received 200 μl of bacterial suspension (10^8^ CFU/ml) daily for 14 consecutive days.

### 2.2 Behavioral tests

#### 2.2.1 Open field test

The open field test was used to assess the locomotor activity of mice. The mice were allowed to be acclimatized to the experimental environment for half an hour before the test. The mice were randomly placed anywhere in the open field test chamber and permitted to investigate freely for 5 min. The ANY-maze automated video system (MED Associates, Georgia, VT, United States) was used to record all the activity of mice through a camera video. The total distances were calculated to investigate the motor function of mice.

#### 2.2.2 Rotarod test

The rotarod test was conducted to evaluate the motor function of mice. Briefly, mice were placed in five separate chambers on a spinning rotarod (Ugo Basile, Comerio, Italy), which can accelerate from 4 rpm to 40 rpm within 5 min. The latency to fall from the rotarod was recorded. All mice are allowed to train for five consecutive days before the final test. Three trials were repeated, and the average latency time was used to analyze the motor function of mice.

#### 2.2.3 Pole test

Briefly, a wooden pole (0.5-m long, 1-cm-diameter) which has a 2-cm-diameter latency was held on a wooden board as its base. Mice were allowed to climb down the pole and the time spent from releasing the mice on the top latency to the one hind limb reaching the base was recorded. All mice are allowed to train 3 times daily for five consecutive days before the final tests. Three trials were performed, and the average time was measured to analyze the motor function of mice.

### 2.3 Real-time quantitative PCR

Extraction of total mRNA was performed using TRIzol reagent (Invitrogen, Carlsbad, CA, USA) and cDNA was synthesized using a Hifair^®^ RT Kit (Yeasen, Shanghai, China). Real-time qPCR was carried out using Hieff UNICON^®^ qPCR SYBR Green Master Mix (Yeasen) with 7500 Real-Time PCR System (Applied Biosystems, Foster City, CA, USA). The results were analyzed using the 2^-△△CT^ method. The primer sequences used in this experiment are shown in **Supplemental Table S1**.

### 2.4 Western blotting

Mice were sacrificed to collect the brain and frozen at -80°C. Brain tissues were lysed, and centrifuged at 12000 rpm and 4°C for 20 min to obtain the total protein in the supernatant. The protein concentrations were estimated by the bicinchoninic acid (BCA) protein assay kit (Thermo Scientific Rockford, IL, USA). Then, the protein was boiled in sodium dodecyl sulfate (SDS)-loading buffer at 95°C for 10 min to denature. The appropriate amount of protein was run on a 10% SDS-polyacrylamide gel electrophoresis (SDS-PAGE), transferred to 0.22 μm polyvinylidene fluoride membranes, and the membranes were blocked with 5% fat-free milk at room temperature for 1 h. The membranes were incubated at 4°C overnight with primary antibodies and incubated at room temperature for 1 h with the HRP-conjugated secondary antibody. Finally, an enhanced chemiluminescence reagent (Thermo Scientific Rockford) was used to develop the blots with a gel image system (Bio-Rad, CA, USA), and the analysis is performed by using ImageJ 1.49v (National Institutes of Health, USA, https://imagej.en.softonic.com/). The following antibodies were used: anti-TH antibody (Cat# ab112, Abcam, Cambridge, MA, USA), anti-β-actin antibody (Cat# 8457S, Cell Signaling Technology, Berkeley, CA, USA), anti-α-syn antibody (Cat# ab212184, Abcam), goat anti-rabbit antibody (Cat# ab6721, Abcam),.

### 2.5 Immunohistochemistry and immunofluorescence

For immunohistochemistry (IHC), the brains were separated in PFA solution at 4°C overnight and embedded with 3% agarose in PBS. Coronal sections were gathered using VT1000 vibratome (Leica Biosystems, Wetzlar, Germany) at a thickness of 30 μm throughout the brain, including the SNpc and striatum and every 3 sections were analyzed. 3% H_2_O_2_ was used to block endogenous peroxidase activity for 10 min at room temperature and sections were blocked with 10% donkey serum for 10 min. For immunofluorescence (IF), sections were blocked with 10% donkey serum for 30 min. Then, the sections were incubated with primary antibody. The sections were incubated with an HRP-conjugated anti-rabbit antibody, stained using the DAB kit (Vector Labs, Carlsbad, CA, USA), and were incubated overnight at 4°C. Fluorescein was combined with a fluorescent secondary antibody. Images were gathered using a fluorescence microscope (Olympus, Tokyo, Japan). The quantifications were performed using ImageJ. The following antibodies were used: anti-Iba1 antibody (Cat# ab5076, Abcam), anti-GFAP antibody (G3893, Sigma-Aldrich, St Louis, MO, USA), anti-α-syn antibody (Cat# ab212184, Abcam), anti-pSer129-α-syn antibody (Cat# ab51253, Abcam), Goat anti-rabbit antibody (Cat# ab6721, Abcam), donkey anti-mouse antibody (Cat# A21203, Invitrogen, Carlsbad, CA, USA), donkey anti-goat antibody (Cat# A32758, Invitrogen).

### 2.6 Nissl staining

Brain sections were made according to the above methods. The sections were washed twice in sterile water, stained with 0.1% cresyl violet (Sangon Biotech) solution buffer for 10 min, washed twice again. Images were obtained by using phase-contrast microscope (Olympus).

### 2.7 Thioflavin-S staining

Thioflavin S staining was performed to measure the aggregation of α-syn. Briefly, the sections were stained with 10 mg/ml thioflavin-S solution (CAT# HY-D0972, MCE, NJ, Monmouth Junction, USA) for 5 min, washed twice. The sections were mounted on slides and images were gathered using a fluorescence microscope (Olympus). The percentage of thioflavin-S-positive areas was calculated to evaluate α-syn aggregation.

### 2.8 Fecal collection and 16S rRNA sequencing analysis

Fecal pellets were gathered 3 days before sacrificed, allowing mice to empty sterile cages and collected immediately in sterile tubes at dry ice and then stored at -80°C. Microbial genomic DNA isolation was performed using a E.Z.N.A.^®^ Soil DNA Kit (Omega Bio-tek, Norcross, GA, USA) The concentration of DNA was obtained by a nanodrop (Thermo Fisher Scientific), and the quality was assessed using agarose gel electrophoresis. The regions V3-V4 of bacterial 16S rRNA gene were amplified using the primers: forward primer (338F 5′-ACTCCTACGGGAGGCAGCA-3′) and the reverse primer (806R 5′-GGACTACHVGGGTWTCTAA-3′). The amplicons were further purified by the AxyPrep DNA Gel Extraction Kit (Axygen Biosciences, Union City, CA, USA) and quantified by QuantiFluor™-ST (Promega, USA), then sequencing was performed on a (2 × 300) bp paired-end Illumina MiSeq platform.

Alpha diversity analysis were performed through Chao and Shannon using QIIME (Version 3.3.1). The differences in the complexity of bacteria between samples were analyzed using the principal coordinate analysis (PCoA) based on Adonis and analysis of similarity (ANOSIM) on QIIME. Linear discriminant analysis (LDA) coupled effect size measurements (LEfSe) analysis was conducted to compare the differences abundances of microbiota at different taxon levels between the groups. Heatmap was plotted using R package 3.5.1. Kyoto Encyclopedia of Genes and Genomes (KEGG) and orthologous groups of proteins (COG) analysis was used to predict the abundances of functional categories by phylogenetic investigation of communities by reconstruction of unobserved state (PICRUSt) software (http://picrust.github.io/picrust/).

### 2.9 Serum samples preparation and UHPLC-MS/MS analysis

On day 28, mice were sacrificed and the whole blood samples were carefully collected, then were kept at room temperature for 1 h to ensure complete clotting. The samples were centrifuged at 5000 rpm and 4°C for 20 min to obtain serum. Serum content (40 mg) was immersed with extract solution, homogenized for 4 min, and sonicated for 5 min in the ice-water bath. Then the samples were centrifuged at 12,000 rpm for 5 min. 200 μL of the extracts were transferred to a fresh tube and derivatized 37 °C. The dried samples were reconstituted and centrifuged to obtain 75 μL of supernatant for LC/MS analysis. The samples were separated on a 1290 Infinity series UHPLC System (Agilent Technologies), equipped with UPLC BEH Amide column (2.1 mm × 10 mm, 1.7 μm). The mobile phase consisted of 25 mmol/L ammonium acetate in water (A) and 25 mmol/L ammonia hydroxide in acetonitrile (B). The elution gradient was as follows: 0~0.5 min, 95%B; 0.5~7.0 min, 95%~65% B; 7.0~8.0 min, 65%~40% B; 8.0~9.0 min, 40% B; 9.0~9.1 min, 40%~95% B; 9.1~12.0 min, 95% B. MS/MS spectra were acquire using the TripleTOF 6600 mass spectrometer (AB Sciex) based on an information-dependent acquisitio. MS data acquisition and quantitation were carried out using R package XCMS (version 3.2). Principal component analysis (PCA) was performed to identify the differences in metabolic patterns among 3 groups. Variable importance of projection (VIP) of each metabolite was obtained based on the orthogonal partial least square discriminant analysis (OPLS-DA) model. Relative contents of metabolites between two groups were calculated using two-tailed Student’s *t* test. Substances with *p* < 0.05 and *VIP* > 1 were identified as differentially altered metabolites among 3 groups. Metabolic pathway enrichment analysis was performed using topology analysis based on KEGG database. The significance of pathway was evaluated using the impact value created from topology analysis.

### 2.10 Histological analysis

Hematoxylin-eosin (H&E) staining was performed according to the manufacturer’s instruction (Kim et al., 2012) as follows: crypt architecture (normal, 0-severe crypt distortion with loss of entire crypts, 3), degree of inflammatory cell infiltration (normal, 0; dense inflammatory infiltrate, 3), muscle thickening (base of crypt sits on the muscularis mucosae, 0; marked muscle thickening, 3), crypt abscess (absent, 0; present, 1), and goblet cell depletion (absent, 0; present, 1). The total histologic scores were the sum of each sample score.

### 2.11 Statistical analysis

All results are analyzed by GraphPad Prism 7 software (https://www.graphpad.com/) using one-way ANOVA. Pearson’s correlation analysis was used to examine the correlation between levels of gut bacteria, peripheral blood metabolites, and PD associated results. The data shown as mean ± SEM and the statistical difference between groups are represented as *p* values, where *p* < 0.05 were considered statistically significant. Significance was performed as *(*p*-value < 0.05), **(*p*-value < 0.01), ***(*p*-value < 0.001), ****(p-value <0.0001).

## 3 Results

### 3.1 The administration of CUR ameliorated motor impairments and dopaminergic neuronal loss in MPTP-induced PD mice.

**Figure 1:**
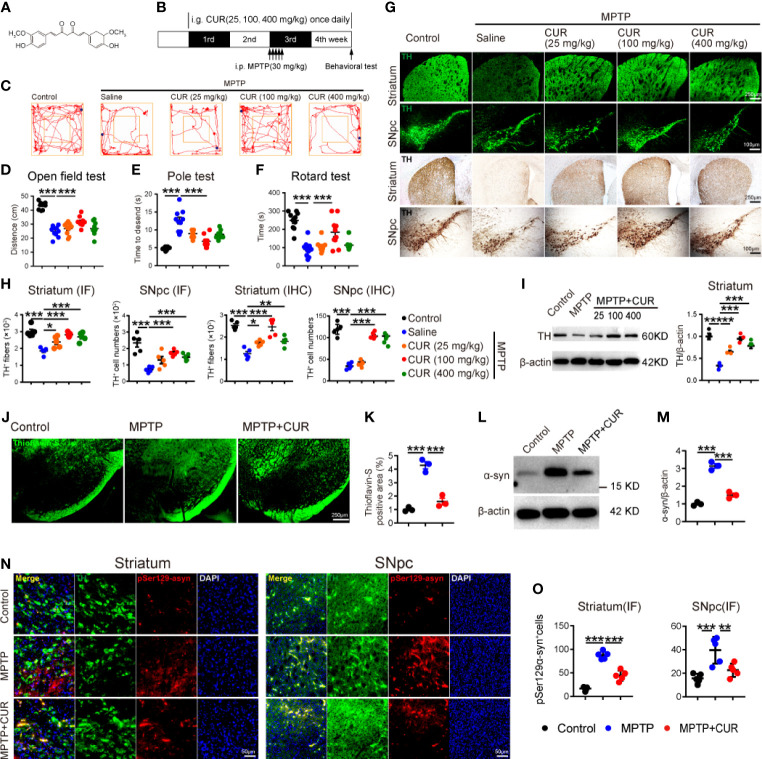
CUR administration prevented motor impairments, dopaminergic neuronal loss and α-synuclein aggregation in MPTP-induced PD mice. Male C57BL/6 mice were received intragastric administration of CUR (25,100,400 mg/kg) once a day for 4 weeks. On day 15, mice were treated with 200 μL saline containing MPTP (30 mg/kg) or 200 μL saline *via* intraperitoneal injection every day for a total of 5 times to establish PD mice model. On day 28, behavioral tests were performed to evaluate the motor function and the mice were sacrificed to determine the pathology of PD by immunohistochemistry, immunofluorescence and immunoblot. **(A)** The molecular structure of CUR. **(B)** The experimental design of CUR intervention in MPTP-induced PD mice. **(C, D)** Representative traces and quantification of the open field test among MPTP treated mice, CUR treated mice and controls (n = 10). Bluepoint: starting position; Redpoint: ending position. **(E, F)** Quantification of performance in the pole test and the rotarod test among 5 groups (n = 10). **(G)** Representative immunofluorescence (upper) and immunohistochemistry (lower) images of TH-positive fibers and neurons in the striatum and SNpc among 5 groups. **(H)** Quantification of TH-positive fibers and neurons in the striatum and SNpc (n = 5). **(I)** Representative bands of TH protein in striatum of one hemisphere determined by WB and quantification of TH expression among 5 groups (n = 3). **(J)** Representative thioflavin-S staining images of α-syn-positive aggregations in the SNpc. **(K)** Quantification of the percentage of thioflavin-S stained area in the SNpc among MPTP treated mice, CUR (100 mg/kg) treated mice and controls (n = 3). **(L, M)** Representative bands of α-syn protein in striatum of one hemisphere determined by WB and quantification of α-syn expression among MPTP treated mice, CUR (100 mg/kg) treated mice and controls (n = 3). **(N)** Representative immunofluorescence images of pSer129α-syn-positive cells in the striatum and SNpc among 3 groups. **(O)** Quantification of pSer129α-syn-positive cells in the striatum and SNpc among 3 groups (n = 5). Data are expressed as mean ± SEM and representative results are one of the independent experiments. All statistical differences were tested using one-way ANOVA in **(C–E, F, G, I, K, M, O)**. Quantification of TH-positive fibers and neurons, α-syn-positive area, pSer129-α-syn-positive cells was performed by ImageJ. *p < 0.05, **p < 0.01, ***p < 0.001, ****p < 0.0001.

**Figure 1A** shows the molecular structure of CUR. First, we verified the dose-dependent effects of CUR using a low-dose, middle-dose and high-dose (25, 100 and 400 mg/kg, respectively) for 4 weeks in the mouse model of PD induced by MPTP, as described previously (**Figure 1B**) (Liu H et al., 2020). MPTP exerts a neurotoxic effect that specifically destroys DA neurons in the SNpc Given that we sought to confirm the role of CUR on the gut microbiota, we administered MPTP *via* the intragastric route. Three behavioral tests were used to determine the motor function of experimental mice. We detected a significant reduction in the total distance travelled by MPTP mice in the open field test, an increase in the descent-time in the pole test, and a decrease in the latency time in the rotarod test when compared with control mice; 100 mg/kg of CUR had the most significant reversal effect on motor impairments (**Figures 1C–F**). No significant improvements were observed in mice receiving 25 mg/kg or 400 mg/kg of CUR when compared with MPTP mice. Collectively, these results revealed that the dose of CUR is critical for its protective effect on PD. Next, considering that DA neuronal loss is the typical pathological characteristic of PD, we analyzed DA neurons in the SNpc-striatum system by IF, IHC and western blotting. DA neurons are marked with tyrosine hydroxylase (TH), which is a rate-limiting enzyme in dopamine synthesis and is recognized as a marker of DA neurons (Sampson et al., 2016). A significant increase in the number of DA cells in the SNpc and their projecting fibers in the striatum were detected with all three doses of CUR mice; the 100 mg/kg CUR group performed better than the other two CUR groups when compared with the MPTP mice (**Figures 1G, H**). Western blotting of TH protein further suggested that the 100 mg/kg dose of CUR was better than the other two groups in terms of the levels of TH (**Figure 1I**). Therefore, this data strongly suggested that the administration of CUR at a dosage of 100 mg/kg can prevent motor impairments and DA neuronal loss in MPTP-induced PD mice. Thus, all subsequent experiments were performed with a dose of 100 mg/kg CUR. Nissl staining showed that DA neuronal loss in the SNpc and striatum was significantly lower in MPTP mice than in control mice; the administration of CUR reversed these effects (**Figures S1A, B**). Taken together, these results indicate that CUR can alleviate MPTP-induced motor impairments and DA neurons neurodegeneration in the SNpc-striatum axis.

### 3.2 The administration of CUR prevented the aggregation of α-synuclein in the MPTP-induced mice model of PD.

The accumulation of Lewy bodies consisting of filamentous aberrant aggregations of α-syn is recognized as another specific pathological hallmark of PD (Sampson et al., 2016). To investigate the effect of CUR treatment on the α-syn deposition, thioflavin-S staining and immunoblotting were performed. The results demonstrated that thioflavin-S-positive area in SNpc was notably elevated in MPTP treated mice while CUR administration sharply reduced the percentage of stained area (**Figures 1J, K**). Consistently, the level of α-syn in striatum also upregulated in MPTP mice and decreased after CUR treatment (**Figures1L, M**). These findings suggested that CUR intervention inhibited α-syn aggregation in MPTP treated mice. Then, serine 129 phosphorylated α-syn (pSer129α-syn) was reported as the main fibril forms of α-syn in LB (Goulding et al., 2020). Hence, IHC and IF of pSer129α-syn were conducted to investigate the effect of CUR administration on pSer129α-syn. Small accumulations of pSer129α-syn immunostaining were observed in MPTP mice when compared with control mice in the SNpc (**Figures 1N, O**, **Figures S1C, D**). Conversely, a lower number of pSer129α-syn-positive inclusions were found in CUR-treated mice when compared with MPTP-treated mice in the SNpc and striatum. These findings suggest that CUR prevented α-syn aggregation in the SNpc-striatum axis of MPTP-induced PD mice.

### 3.3 The administration of CUR suppressed glial cell activation and neuroinflammation in the SNpc and striatum in an MPTP-induced mouse model of PD.

**Figure 2:**
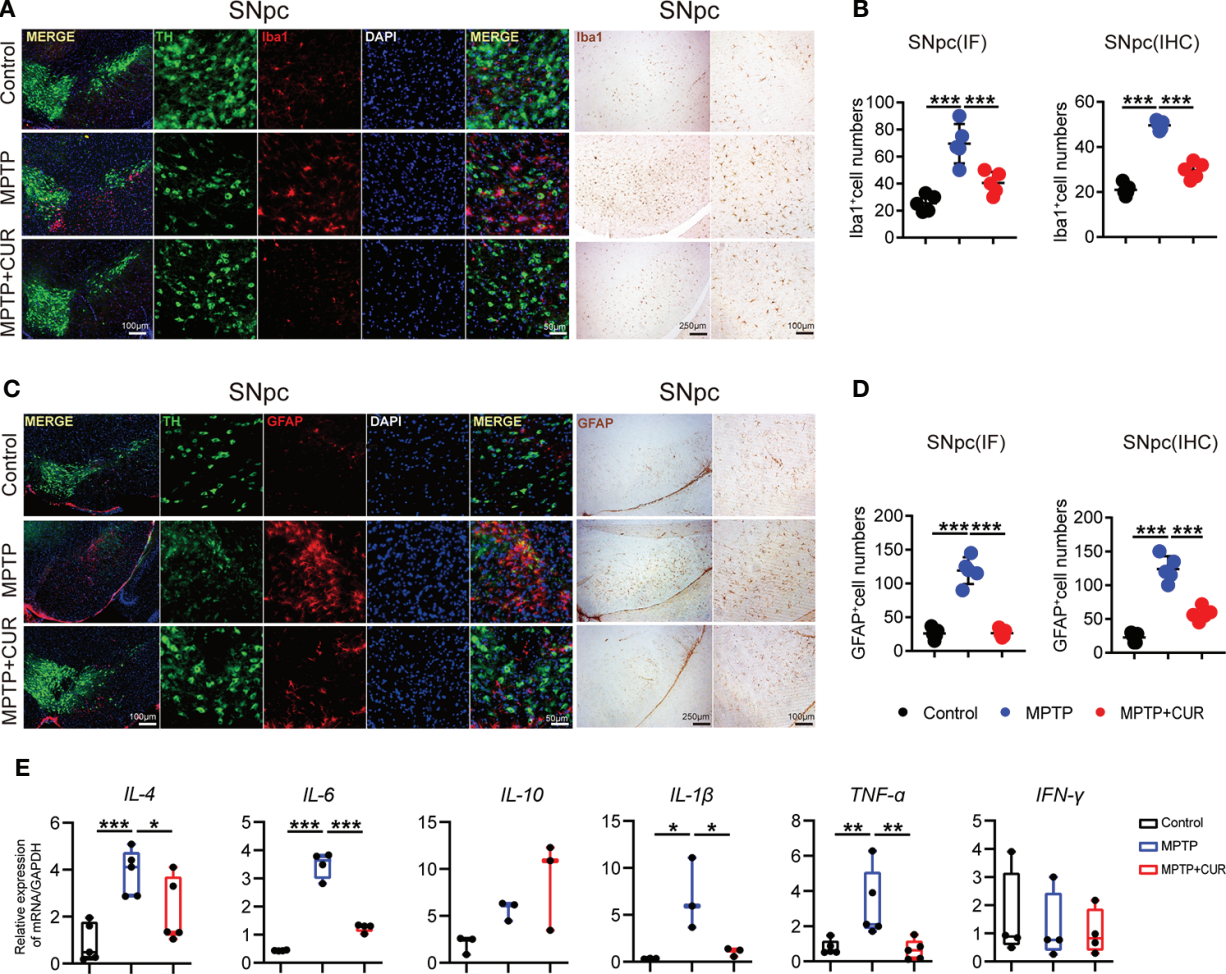
CUR administration suppresses glial cells activation in SNpc in MPTP-induced mice model. Male C57BL/6 mice were received intragastric administration of CUR (100 mg/kg) once a day for 4 weeks. On day 15, mice were treated with 200 μL saline containing MPTP (30 mg/kg) or 200 μL saline *via* intraperitoneal injection every day for a total of 5 times to establish PD mice model. On day 28, the mice were sacrificed to determine the reactive glial cells of PD by immunohistochemistry and immunofluorescence. **(A)** Representative immunofluorescence images of nuclei (DAPI, blue), dopaminergic neurons (TH, green), and microglia (Iba1, red) in the SNpc among MPTP treated mice, CUR treated mice and controls. **(B)** Quantification of Iba1 positive cells in the SNpc using immunofluorescence and immunohistochemistry staining among 3 groups (n = 5). **(C)** Representative immunofluorescence staining of nuclei (DAPI, blue), dopaminergic neurons (TH, green), and astrocytes (GFAP, red) in the SNpc among MPTP treated mice, CUR treated mice and controls. **(D)** Quantification of GFAP positive cells in the SNpc using immunofluorescence and immunohistochemistry staining (n = 5) among 3 groups. **(E)** The expression of pro-inflammatory genes in the striatum among 3 groups (n = 3-6). Data are expressed as mean ± SEM and representative results are one of the independent experiments. All statistical differences were tested using one-way ANOVA in (B, D, E) Quantification of Iba1-positive cells, GFAP-positive cells was performed by ImageJ. *p < 0.05, **p < 0.01, ***p < 0.001.

According to previous researches, the degeneration of DA cells in MPTP-induced mice is always accompanied by glial activation (Bloem et al., 2021). Thus, we sought to investigate microglial and astrocyte activation in the SNpc and striatum. Microglia undergo remarkable morphological alterations upon activation, exhibiting an increased cell body number and diameter or fewer branches. Iba1 (as a marker of microglia) immunostaining in the striatum and SNpc revealed that CUR administration prevented MPTP-induced microglia activation when compared with control mice; quantitative analysis of microglia is shown (**Figures 2A, B**; **Figures S2A, B**). Furthermore, activated microglial morphology was clearly evident in MPTP-treated mice but not in control mice or CUR-treated mice. Analysis of GFAP (a marker of astrocytes) immunostaining also revealed significant down-regulation in the SNpc-striatum axis after CUR treatment when compared with MPTP mice (**Figures 2C, D**; **Figures S2C, D**). Collectively, these results illustrate that CUR treatment significantly attenuated PD-related glial cell activation both in the SNpc and the striatum.

It was reported that microglia play an essential role in neuroinflammation of PD *via* production of proinflammatory cytokines, such as tumor necrosis factor (TNF)-α, interleukin (IL)-1β, IL-4, and IL-6 (Hickman et al., 2018). Therefore, we detected expressions of some neuroinflammatory mediators in striatum of mice using real-time quantitative PCR. The mRNA levels of *IL-4*, *IL-6*, *IL-1β*, *TNF-ɑ* were elevated in MPTP treated mice, while they were sharply down-regulated after CUR intervention (**Figure 2E**). Taken together, CUR treatment prevented the MPTP induced activation of glial cells and neuroinflammation.

### 3.4 The administration of CUR alters the profile of the gut microbiota in an MPTP-induced mouse model of PD

**Figure 3:**
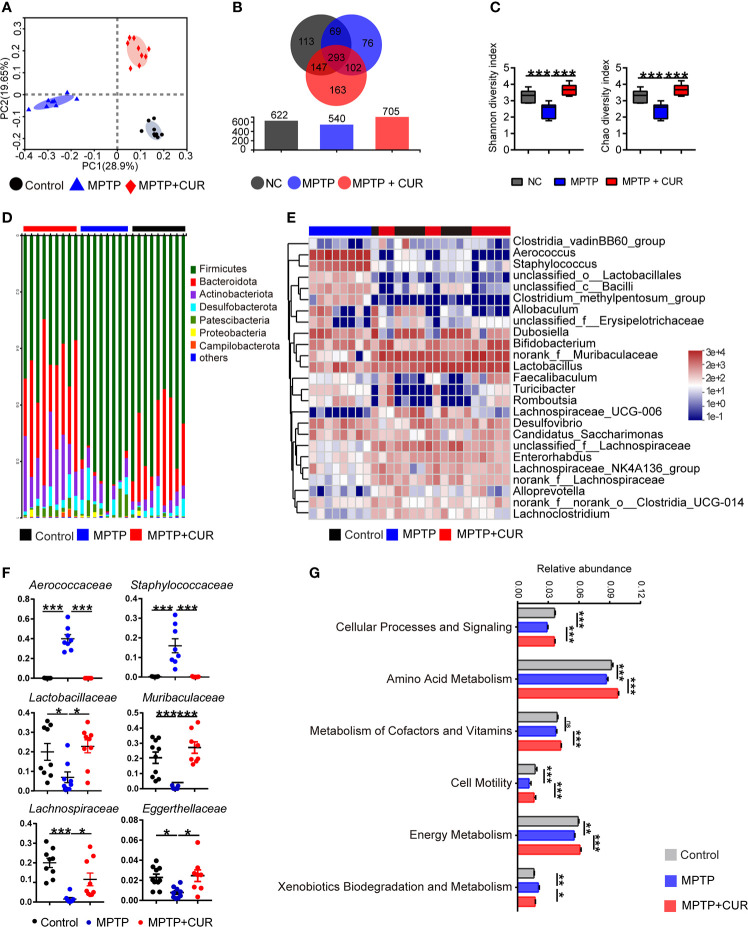
CUR administration alters the profile of gut microbiota in MPTP-induced PD mice model. Male C57BL/6 mice were received intragastric administration of CUR (100 mg/kg) once a day for 4 weeks. On day 15, mice were treated with 200 μL saline containing MPTP (30 mg/kg) or 200 μL saline *via* intraperitoneal injection every day for a total of 5 times to establish PD mice model. On day 28, fecal pellets were collected from the mice to detect the gut microbiota by using 16S rRNA sequencing (n = 8-10). **(A)** The PCoA plot of MPTP treated mice, CUR treated mice and controls. **(B)** Venn diagram of shared and unique OTUs among 3 groups. The histogram represents the number of OTUs of each group. **(C)** The Shannon index and Chao index of MPTP treated mice, CUR treated mice and controls. **(D)** Composition of gut bacteria at phylum level shows a decrease in the abundance of *Bacteroidota* and an increase in abundance of *Firmicutes* in MPTP group compared with the other two groups by a bar plot. **(E)** Composition of top 25 genera at genus level between 3 groups using a heatmap. **(F)** Relative abundances of *Aerococcus*, *Staphylococcaceae*, *Lactobacillaceae*, *Muribaculaceae*, *Lachnospiracea*, *Eggerthellaceae* at family level using scatter points diagram. **(G)** Relative abundances of KEGG pathway level 2 classifications between MPTP mice and CUR mice using a bar plot. Data are expressed as mean ± SEM. All statistical differences were tested using one-way ANOVA in **(C, F, G)** *p < 0.05, **p < 0.01, ***p < 0.001n.s, not significant.

Given that CUR has been found to modulate the gut flora in some nervous system diseases, we analyzed the microbial composition of fecal samples in three experimental groups using 16S rRNA sequencing (Huang et al., 2021). First, principal coordinate analysis (PCoA) revealed that the gut microbial composition of MPTP-induced PD mice was significantly different from that of the control and CUR group, as revealed by Adonis test (*p* = 0.001; **Figure 3A**). Meanwhile, similar results were found using clustering and phylogenetic tree analysis (**Figure S3A**). Then, Venn diagram analysis further showed that CUR and control mice showed significantly higher numbers of operational taxonomic units (OTUs) when compared with the MPTP mice (**Figure 3B**). Furthermore, alpha-diversity analysis, which was conducted to evaluate the richness and diversity of the bacterial species, revealed a significantly reduced Shannon index (*p* = 0.001) and Chao index (*p* = 0.037) in MPTP-treated mice when compared to the control mice. The CUR-treated group showed significantly higher indices (Shannon index: *p* = 0.000; Chao index: *p* = 0.002) respectively, thus suggesting that CUR treatment significantly restored the MPTP induced reduction in richness and diversity of intestinal bacteria (**Figure 3C**). Alterations of the GM at the phylum, family and genus levels are shown (**Figures 3D, E**; **Figures S3, B**). Notably, at the family level, we identified the top six alterations across the three groups. (**Figure 3F**). Linear discriminant analysis (LDA) coupled with effect size measurements, was performed to confirm the core bacterial genera across the three groups (*p*<0.05, LDA score >4.0), **Figures S3C, D**). Analysis showed that 10 bacterial taxa were enriched in the control group, 16 taxa in the MPTP group and 19 taxa in the CUR group (LDA score >4.0; S3D).

To better interpret the functions of these significantly altered bacteria, KEGG pathways and COG analysis were performed based on the PICRUSt. As shown in **Figure 3G**, amino acid metabolism, cellular processes, signaling and cellular processes were significantly enriched in the control group and CUR mice when compared with MPTP mice. Xenobiotic biodegradation and metabolism were enriched in the MPTP group. COG analysis showed similar results in that the amino acid metabolism pathway was enriched in the CUR and control groups when compared with the MPTP group (**Figure S3E**).

### 3.5 The gut microbiota mediated the neuroprotective effects of CUR in an MPTP-induced mouse model of PD

**Figure 4:**
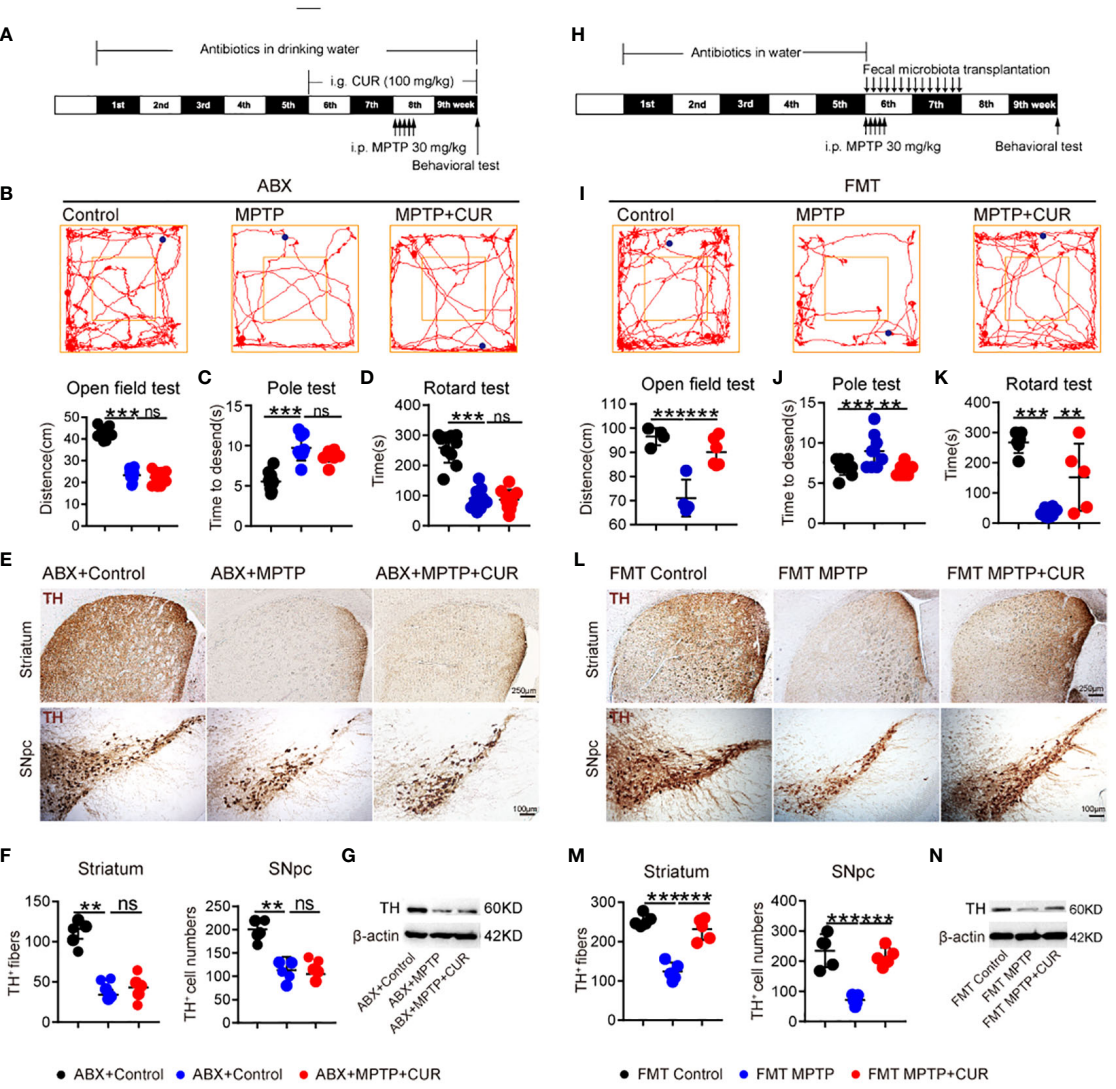
Antibiotic pretreatment (ABX) and fecal microbiota transplantation (FMT) validated that the gut microbiota mediated the neuroprotective effect of CUR in the MPTP-induced mice model. For the ABX treatment experiment, male C57BL/6 mice were received intragastric administration of CUR (100 mg/kg) once a day for 4 weeks starting from day 36. On day 50, mice were treated with 200 μL saline containing MPTP (30 mg/kg) or 200 μL saline *via* intraperitoneal injection every day for a total of 5 times to establish PD mice model. Mice were received water containing antibiotic solution or regular drinking water alone from the beginning to the end. For the FMT experiment, mice were received water containing antibiotic solution or regular drinking water alone from day1 to day 35. On day 36, FMT administration was performed once a day for 2 weeks. On day 63, behavioral tests were performed to evaluate the motor function and the mice were sacrificed to determine the pathology of mice by immunohistochemistry and immunoblot. **(A)** The experimental design for ABX treatment and 100 mg/kg CUR administration in MPTP-induced PD mice. **(B)** Representative traces and quantification of the open field test among ABX MPTP treated mice, ABX MPTP+CUR treated mice and ABX controls (n = 10). Bluepoint: starting position; Redpoint: ending position. **(C, D)** Quantification of performance in the pole test and the rotarod test among 3 groups (n = 10). **(E)** Representative immunohistochemistry images of TH-positive fibers and neurons in the striatum and SNpc among ABX MPTP treated mice, ABX MPTP+CUR treated mice and ABX controls. **(F)** Quantification of TH-positive fibers and neurons in the striatum and SN (n = 6). **(G)** Representative bands of TH protein in striatum of one hemisphere determined by WB among 3 groups (n = 3). **(H)** The experimental design for FMT experiment. **(I)** Representative traces and quantification of the open field test among FMT MPTP treated mice, FMT MPTP+CUR treated mice and FMT controls. Bluepoint: starting position; redpoint: ending position. **(J, K)** Quantification of performance in the pole test and the rotarod test among 3 groups (n = 6). **(L)** Representative immunohistochemistry images of TH-positive fibers and neurons in the striatum and SNpc among FMT MPTP treated mice, FMT MPTP+CUR treated mice and FMT controls. **(M)** Quantification of TH-positive fibers and neurons in the striatum and SN (n = 6). **(N)** Representative bands of TH protein in striatum of one hemisphere determined by WB among 3 groups (n = 3). Data are expressed as mean ± SEM and representative results are one of the independent experiments. All statistical differences were tested using one-way ANOVA in **(B–D, F, I, J, K, M)**. Quantification of TH-positive fibers and neurons was performed by ImageJ. **p < 0.01, ***p < 0.001, n.s., not significant.

Next, we attempted to confirm whether gut bacteria are critical for the neuroprotective effect of CUR on PD. We established a mouse model and eliminated the gut microbiota with an antibiotic cocktail water four weeks prior to the administration of CUR (**Figure 4A**). Microbes-depletion blocked the CUR-induced improvements in motor function when compared with ABX-MPTP-induced mice (**Figures 4 B–D**). Next, we explored whether microbes depletion influenced the effect of CUR on the loss of DA neurons in MPTP-induced mice. Immunostaining of TH in the SNpc and striatum revealed that the effect of CUR on the attenuation of DA neuron loss was eliminated by microbes-depletion in MPTP mice (**Figure 4E, F**; **Figures S4A, B**). Western blotting of TH in the striatum further confirmed the blocking effect of gut bacteria depletion on CUR-induced protection (**Figure 4G**; **Figure S4C**).

To further confirm the gut flora-mediated neuroprotective effects of CUR on PD, we conducted fecal microbiota transplantation (FMT) from control mice, MPTP mice, and CUR mice into ABX-pretreated mice for 2 weeks (**Figure 4H**). The FMT MPTP mice exhibited poor behavioral performance when compared to the FMT Control and FMT MPTP + CUR mice (**Figures 4I–K**). IF, IHC and western blotting confirmed these results (**Figures 6L–N**; **Figures S4D–F**). The improved motor performance and increased number of DA neurons of mice harboring microbiota from MPTP+CUR mice when compared to MPTP mice demonstrated that the gut microbiota could contribute to the benefits conferred by CUR on motor dysfunction in PD mice. Therefore, both antibiotic treatment and FMT experiments demonstrated that the gut flora is required for the CUR-mediated protection of PD.

The modulation of microglia activation plays an important role in CUR-associated neuroprotection on PD mice, we further examine whether CUR-associated gut microbes could also have regulatory effect on microglia activation. Immunostaining of Ibal in the SNpc showed that the numbers of Ibal-positive cells was remarkably up-regulated in FMT MPTP mice and was sharply decreased in MPTP+CUR mice (**Figures S5A, B**), suggesting that the gut flora transplanted from CUR treated mice is able to mimic the anti-inflammatory effects of CUR.

### 3.6 The administration of CUR promoted PD-associated histological features in the colons of an MPTP-induced mouse model of PD

Since the gut microbiota is crucial to CUR-mediated neuroprotection in PD, we further assessed histological alterations in the colons of the three groups of mice by H&E staining. We observed a disordered arrangement of intestinal epidermal cells and the infiltration of massive inflammatory cells in the colon, cecum, and rectum, manifesting as deleterious intestinal changes in the MPTP mice when compared with control mice; CUR treatment reversed these changes (**Figures S6A, B**). Next, we examined the length of the colon and analyzed GI function to confirm the effect of CUR administration on the gut. We found that the length of the colon remained unchanged following CUR administration (**Figure S6C**). However, the number of fecal pellets produced over 15 min increased remarkably, thus demonstrating that GI function was improved by CUR intervention (**Figures S6D, E**). Collectively, these results clearly demonstrated that CUR treatment led to an obvious improvement in MPTP-induced colon changes.

### 3.7 The administration of CUR changed the profiles of circulating metabolites in an MPTP-induced mouse model of PD

**Figure 5:**
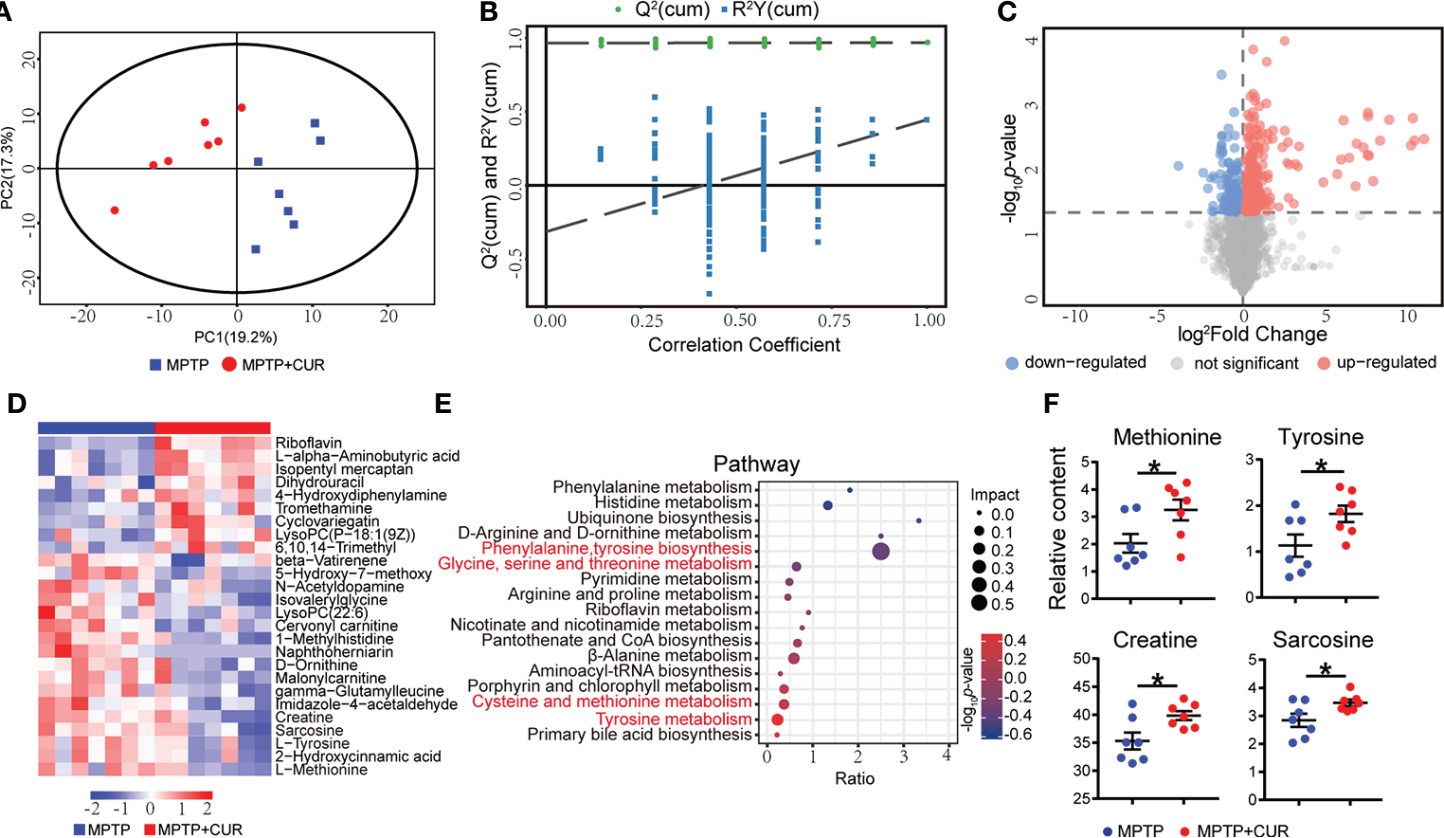
CUR administration changes profiles of the circulating metabolites in MPTP-induced PD mice model. Male C57BL/6 mice were received intragastric administration of CUR (100 mg/kg) once a day for 4 weeks. On day 15, mice were treated with 200 μL saline containing MPTP (30 mg/kg) or 200 μL saline *via* intraperitoneal injection every day for a total of 5 times to establish PD mice model. On day 28, serum was collected from the mice to detect the metabolism by using UHPLC-MS (n = 7). **(A)** The PCoA plot of serum metabonomic between MPTP group and CUR group. **(B)** OPLA-DA analysis of the serum metabonomic between MPTP group and CUR group. **(C)** The differentially expressed metabolites between MPTP group and CUR group using a volcano map. (*VIP* value > 1 and *p* value < 0.05). Each dot represents a detected metabolite. **(D)** Relative contents of differentially expressed metabolites between MPTP and CUR group using a heat map. **(E)** Comparison of KEGG pathway between the MPTP and CUR group using a bobble plot. **(F)** Relative contents of tyrosine, methionine, sarcosine, creatine between MPTP and CUR group. Data are expressed as mean ± SEM. Statistical differences were tested using unpaired two-tailed Student’s *t* test in **(F)** *p < 0.05.

Since metabolites are considered to be pivotal mediators of host-microbiota communication, we hypothesized that gut flora-modulated metabolites may take part in CUR neuroprotection in PD. Therefore, we performed non-targeted metabolomic profiling of serum across the three groups of mice. PCA plot and OPLA-DA showed that the MPTP mice could be easily distinguished from the CUR-treated mice and controls (**Figures 5A B**, **Figures S7A, B**). A total of 4672 metabolites were identified among 3 groups. After analysis, 392 differentially expressed metabolites showed significant changes when compared between the MPTP and CUR mice (**Figure 5C**) and 665 differentially expressed metabolites between the MPTP and control mice (**Figure S7C**). The specific alterations of these differential metabolites are shown (**Figure 5D**; **Figure S7D**; **Supplemental Table S2)**

KEGG analysis was used to identify and map metabolites; results showed that the differential metabolic pathways between the MPTP and CUR mice included phenylalanine, tyrosine and tryptophan biosynthesis, tyrosine metabolism, cysteine and methionine metabolism (**Figure 5E**). In particular, the levels of tyrosine, methionine, creatine and sarcosine were significantly lower in the MPTP mice when compared to the CUR mice (**Figure 5F**). Meanwhile, the KEGG analysis of MPTP mice and controls are showed (**Figure S7E**).

### 3.8 Correlations between changes in gut microbiota profile, serum metabolism alterations and PD-related pathological results

**Figure 6:**
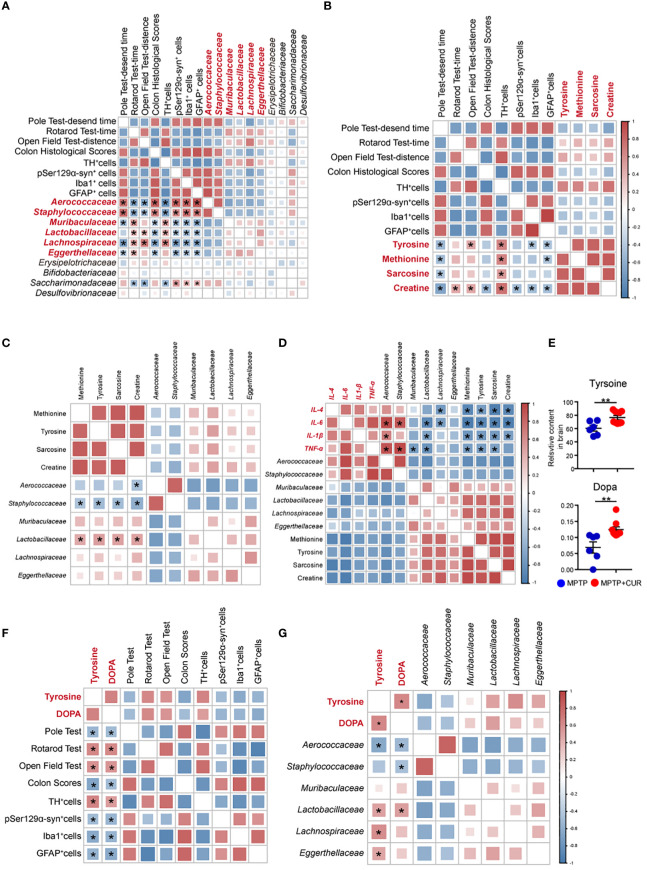
Correlations between changes in gut microbiota profile, serum metabolism alterations and PD-related pathological results. **(A)** Heatmap of Pearson’s correlation coefficients between the abundances of top 10 taxa at family level and PD associated results. Bacterial groups in red represent the top 6 altered taxa. **(B)** Heatmap of Pearson’s correlation coefficients between the levels of differentially-altered serum metabolites and PD associated results. **(C)** Heatmap of Pearson’s correlation coefficients between the abundances of 6 altered bacteria and PD associated results. **(D)** Heatmap of Pearson’s correlation coefficients between the expression of differentially-altered inflammatory cytokines and levels of pivotal gut bacteria and metabolites. **(E)** The relative contents of tyrosine and dopa in the brain. **(F)** Heat map of Pearson’s correlation coefficients between the brain levels of tyrosine and dopa and PD associated results. **(G)** Heat map of Pearson’s correlation coefficients between the tyrosine and dopa levels of brain and the key altered gut microbiota taxa. Data are expressed as mean ± SEM. Statistical differences were tested using Pearson’s correlation analysis. *p < 0.05; **p< 0.01.

To better interpret the effects of the significant bacterial alterations in the neuroprotective effects of CUR in PD, we performed correlation analysis between the relative abundances (RAs) of the top 10 flora and PD-associated motor function and pathological results of mice. Among them, top 6 altered bacteria were found to have significant correlation with PD-associated results. As shown in **Figure 6A**, the RAs of *Lactobacillaceae*, *Lachnospiraceae*, M*uribaculaceae*, *Eggerthellaceae* showed strong positive correlations with PD associated results. Negative correlations were identified between *Aerococcaceae*, *Staphylococcaceae* and PD associated results. Overall, these data demonstrated that gut microbiota is likely to be involved in protective effects of CUR on MPTP mice, thus suggesting the critical role of gut flora in PD pathology. Furthermore, we performed correlation analysis of significantly altered metabolites and PD associated results. The levels of tyrosine, methionine, sarcosine and creatine exhibited a significant positive correlation with PD associated results, thus demonstrating the role of metabolites in CUR-induced neuroprotection in PD (**Figure 6B**). Then, to further investigate the interaction between gut bacteria and metabolites, we correlated the top six altered genera with serum metabolites. Pearson’s correlation analysis identified several significant associations between gut bacteria and serum metabolites. Tyrosine showed strong correlations with *Lactobacillaceae*, *Aerococcaceae* and *Staphylococcaceae* genera. Furthermore, *Staphylococcaceae* was negatively correlated with sarcosine and creatine (**Figure 6C**). To explore the in-depth linkage between gut microbiota-metabolite axis and PD pathogenesis, the correlation analysis between the pivotal gut flora and serum metabolites and the levels of *IL-4*, *IL-6*, *IL-1β*, *TNF-ɑ* was performed. Of note, abundances of *Lactobacillaceae*, *Aerococcaceae* correlated with the levels of *IL-6*, *IL-1β*, *TNF-ɑ*. The levels of tyrosine, methionine and sarcosine had remarkable correlation with expression of *IL-4*, *IL-6*, *IL-1β*, *TNF-ɑ*in the striatum of mice (**Figure 6D**). Taken together, the findings further supported the notion that the essential role of CUR in modulation gut microbiota-metabolism axis, thus contributing to the protective and anti-neuroinflammatory effect of CUR in PD.

It’s worth noting that tyrosine, one of altered metabolites, were recognized as a vital amino acid involved in pathology of PD (Hirayama et al., 2016). Tyrosine is a non-essential amino acid that can be synthesized from phenylalanine and represents an essential component for the production of several important brain neurotransmitters, including dopamine, epinephrine and norepinephrine (Zhang et al., 2022). Moreover, the serum concentration of tyrosine was found to exhibit positive correlations with the motor function of mice and crucial gut taxa. Thus, to uncover the effect of circulating tyrosine on the CNS, we next performed neurotransmitter-targeted metabolomics profiling of brain tissues from mice in the MPTP and CUR groups. Intriguingly, the levels of tyrosine and dopa in the brain also increased following CUR administration (**Figure 6E**). Finally, we carried out correlation analysis between metabolites undergoing significant changes in the brain and alterations in the gut microbiota. The levels of tyrosine and dopa in the brain exhibited a significant and positive correlation with PD associated results (**Figure 6F**). Both *Lactobacillaceae* and A*erococcaceae* showed significant associations with the levels of tyrosine and dopa in the CNS (**Figure 6G**). The detailed statistical results of Pearson’s correlation analysis were presented in **Supplemental Table S3**.

## 4 Discussion

The findings of this study demonstrated that CUR invention ameliorated the motor dysfunction and GI functions of MPTP mice and the gut microbiota is required for beneficial effects of CUR. Furthermore, CUR remarkably upregulated the abundances of *Lactobacillacea* and *Lachnospiraceae* and downregulated abundances of *Aerococcaceae* and *Staphylococcaceae*, as well as elevation in serum levels of tyrosine, methionine, sarcosine and creatine. More importantly, CUR associated alterations in microbiota-metabolite axis is closely correlated with the pathology of PD mice. Of note, CUR activated tyrosine-dopa metabolism, which ultimately contributed to the elevation of dopamine. Taken together, our data provide novel evidence for the role of CUR in modulation of microbiota-metabolism axis involved in PD pathology, and offer new insight into the pathogenesis of PD. Most of all, novel therapeutic strategy based on CUR may exert a desirable effect on the GI disturbances complementary to the current PD treatment.

A large body of cell and animal research has investigated the positive effects of CUR in PD (Qualls et al., 2014; van der Merwe et al., 2017; Sharma and Nehru, 2018; Dehghani et al., 2020; Abrahams et al., 2021). For example, seven weeks of dietary supplementation with CUR were previously shown to prevent the loss of DA neurons in the SNpc and striatum in MPTP mice (He et al., 2015). CUR has also been shown to exert neuroprotective effects in the rotenone-induced PD model and the 6-hydroxydopmine-induced rat model (Motawi et al., 2020; El Nebrisi et al., 2020). Of note, we found that CUR restrains the activation of the glial cells in the SNpc-striatum axis; these findings are similar to those of a previous study involving MPTP mice (He et al., 2015). Furthermore, we demonstrated that the inhibition of α-syn aggregation in the brain contributed to the beneficial effects of CUR; this has also been mentioned in several studies (Pandey et al., 2008; Sharma and Nehru, 2018).

In addition, CUR has been shown to contribute to the prevention of colitis and colon cancer by modulating the community of gut flora, thus reducing intestinal inflammation and improving the function of the intestinal barrier (McFadden et al., 2015). Indeed, CUR-driven alterations of gut flora have been confirmed to play a vital role in multiple diseases. CUR supplementation alters the composition of gut microbiota and CUR metabolism, which contributes to the protective effects of CUR in obesity, diabetes and hypertension (Huang et al., 2021; Islam et al., 2021; Li et al., 2021). Of note, the bidirectional interactions between CUR and gut bacteria have been postulated to trigger the etiology of some neurodegenerative diseases. Importantly, CUR was reported to improve learning and memory abilities, reduce the burden associated with amyloid plaques, alter the abundances of several beneficial bacterial taxa and improve the metabolites of curcumin in mice of Alzheimer’s disease (Sun et al., 2020). However, the specific interaction between CUR and gut microbiota in the pathology of PD has yet to be identified. Thus, we performed 16S rRNA sequencing to investigate the CUR-driven gut flora profiles in MPTP mice.

The PD model utilized in our research was a 5-day MPTP-treated sub-acute mouse; this model was based on previous studies and that this model of PD gives rise to gut microbiota imbalance, glial reactions and the aggregation of α-syn aggregation (McFadden et al., 2015), suggesting that the model is an appropriate tool for our study. The changes of gut microbiota observed in our study were in accordance with other studies of PD (Chen et al., 2020) and at the phylum level, led to an obvious reduction in the abundance of *Bacteroidota* and an increase in the abundance of *Firmicutes*.

Our study found that *Aerococcus* was negatively correlated with behavioral tests and the number of TH-positive cells but positively correlated with colon histological scores, pSer129α-syn-positive cells, Iba1-positive cells, and GFAP-positive cells. The hazardous effect of *Aerococcus* was consistent with previous reports in that is a gram-positive bacterium and is increasingly acknowledged as a cause of human infections (Rasmussen, 2016). Patients with *Aerococcal bacteriuria* are more inclined to suffer from urinary tract infection; this might explain why the high abundacne of *Aerococcus* are highly relevant to the severe pathological score of the colon in our findings. Moreover, *Aerococcus* is believed to cause infective endocarditis and vertebral osteomyelitis (Astudillo et al., 2003; Sunnerhagen et al., 2016), thus demonstrating its systemic pro-inflammatory effect. In the present study, we showed that higher abundance of *Aerococcus* were related to elevated numbers of Iba1-positive and GFAP-positive cells. Yet, the pathogenic mechanisms of *Aerococcus* remain obscure and further research is required. Also, we observed increases of *Staphylococcus* taxa in the MPTP group; this was reversed by CUR pretreatment.

A common form of *Staphylococcus* is *Staphylococcus aureus* (*S. aureus*). In a previous study, CUR prevented *S. aureus*-associated infections and inflammation, including mastitis injury, osteomyelitis and acute lung injury (Zhou et al., 2017). CUR is known to prevent *S. aureus*-associated pathology by attenuating nuclear factor-κB (NF-κB) pathway or the activation of tumor necrosis factor-α (TNF-α) and interleukin-6 (IL-6) (Zhou et al., 2017; Xu et al., 2020). Furthermore, CUR has been shown to prevent neuroinflammation by suppressing secretion of TNF-α or IL-6 (Jin et al., 2018; Drion et al., 2018). In the present study, we found that *Staphylococcus* correlated with the increased number of activated glial cells. CUR has been shown to reverse the resistance of methicillin-resistant *S. aureus*, making this an interesting therapeutic alternative for the clinical treatment of PD (Mun et al., 2014; Freitas et al., 2019).

In agreement with many previous studies, we also found that curcumin treatment increased the abundance of *Lactobacillus* (Xu et al., 2021; Qi et al., 2022). *Lactobacillus* has been proven to be beneficial in previous studies and is known to be markedly reduced in PD mice and PD patients (Perez-Pardo et al., 2017). Previous research has also proven the neuroprotective effects of *Lactobacillus* administration in MPTP- and rotenone-induced PD models as well as PD patients (Liao et al., 2020; Ilie et al., 2021; Lu et al., 2021). In agreement with our results, *Lactobacillus* was previously reported to attenuate MPTP-induced neuroinflammation by preventing glial hyperactivation, and inhibiting the levels of *TNF-α*, and *IL-6* (Liao et al., 2020; Perez Visñuk et al., 2020). Intriguingly, *Lactobacillus*-based probiotics increased the levels of both dopamine and serotonin in the striatum, thus ameliorating the anxiety-like behavior of germ-free mice (Liu WH et al., 2016). In our study, we discovered elevated levels of tyrosine and dopa in the brain; these levels were significantly correlated with the increased abundance of *Lactobacillus* after the administration of CUR in MPTP mice. Thus, it appears that *Lactobacillus* has an essential role in CUR-associated microbiome-host interactions and exerts a neuroprotective function in PD, especially in the DA system. Consistent with many previous studies, our results validated elevated levels of *Staphylococcaceae* (Kusbeci et al., 2009), reduced levels of *Lachnospiraceae* (Keshavarzian et al., 2015) and *Prevotellaceae* (Scheperjans et al., 2015) in PD; however, CUR mice showed the opposite results.

Our present research demonstrated that the abundance of the *Lachnospiraceae* family were significantly reduced in the MPTP group when compared with the control group; this finding has been reported in other studies (Unger et al., 2016); CUR treatment reversed this change. Some members of the *Lachnospiraceae* have the potential to produce short-chain fatty acids which are likely to play a beneficial role in the gut microbiota-brain crosstalk axis (Flint et al., 2015). Moreover, some studies have reported that PD patients taking levodopa-carbidopa intestinal gel possessed fewer *Lachnospiraceae* than controls (Melis et al., 2021), thus suggesting that this flora may be involved in dopamine metabolism. We found that the abundance of *Lachnospiraceae* were positively correlated with the levels of tyrosine in the CNS; this finding was consistent with a previous study (Li et al., 2020). However, the mechanisms by which *Lachnospiraceae* modulate tyrosine metabolism require further research.

Three crucial metabolic pathways involved in CUR intervention were confirmed in our study.

### 4.1 Tyrosine → dopa → dopamine metabolism

Tyrosine constitutes a precursor for the catecholamines (dopamine, norepinephrine, and epinephrine) and is predominantly produced from the essential amino acid phenylalanine by the enzyme phenylalanine hydroxylase. First, tyrosine is converted to dopa by the enzyme TH; this is a rate rate-limiting enzyme. Then, dopa is decarboxylated by aromatic l-amino acid decarboxylase to form dopamine, which plays a vital role in the DA system (Zhang et al., 2022). It has been demonstrated that an elevation of tyrosine concentrations in the brain could rapidly stimulate and increase the synthesis and release of dopa and dopamine (Wurtman et al., 1974); the levels of these agents are also influenced by their peripheral concentrations. Therefore, the increased plasma levels of tyrosine detected in our study is likely to have contributed to the rising levels of tyrosine and dopa in the brain due to the protective effect of CUR on PD.

Furthermore, the reduced levels of tyrosine in the plasma of the MPTP group was in accord with a previous study involving PD patients (Hirayama et al., 2016). Previous studies suggested that the gut flora could regulate the production or metabolism of neurotransmitters, including dopamine, serotonin, or gamma-aminobutyric acid and then affect the expression of neurotransmitter receptors in the brain and GI (Bravo et al., 2011; Strandwitz, 2018; Jadhav et al., 2018; Zheng et al., 2019). Interestingly, our correlation analysis showed that both tyrosine and dopa levels in the brain were significantly related to the abundances of *Aerococcaceae* and *Lactobacillaceae*. However, the specific mechanisms by which gut flora alteration contributes to tyrosine → dopa → dopamine metabolism remains complex and further research is required.

### 4.2 Methionine metabolism

Methionine is an essential sulfur-containing amino acid which acts as the main precursor of cysteine which constitutes the primary source for glutathione (GSH) synthesis (Martínez et al., 2017). Our present results showed that the levels of methionine were decreased in the MPTP group and increased after treatment with CUR. Methionine plays an essential role in lipid metabolism, the innate immune system and oxidative stress (Martínez et al., 2017). Specifically, methionine participates in the oxidative stress response *via* methionine catabolism, thus leading to the production of GSH, a low-molecule-weight antioxidant (Blachier et al., 2007). Furthermore, methionine modulates the sulfoxide reductase system to reduce ROS accumulation and ROS levels, thus leading to the stimulation of GSH synthesis (Liu et al., 2008). It was previously reported that methionine protects against the loss of DA neurons and oxidative stress in the mitochondria of the 6-OHDA PD model by modulating nuclear factor erythroid 2–related factor 2 signaling and antioxidant enzymes (Catanesi et al., 2021). Interestingly, longitudinal plasma metabolome profile traces of 30 PD patients and 30 matched controls previously suggested that methionine levels in the PD group were significantly reduced during follow-up; even the PD group show higher methionine levels at baseline (Hertel et al., 2019). Given the severe oxidative stress in PD, our finding might be explained by the higher requirement for generating the antioxidant glutathione.

### 4.3 Sarcosine and creatine metabolism

Sarcosine is a natural amino acid found in food and human body tissues and is known as a precursor and metabolite of glycine (Curtis, 2019). In our study, we found that the levels of sarcosine and creatine were reduced in MPTP mice but significantly increased after CUR treatment. Sarcosine acts as an endogenous glycine transport I inhibitor and restrains glycine uptake, thus leading to an increase of synaptic glycine concentration to enhance N-methyl-D-aspartate (NMDA) receptor function (Javitt, 2009). The NMDA receptor modulates crucial functions related to learning and cognition and the synaptic plasticity of neurodegenerative diseases (Kalia et al., 2008). Based on this, sarcosine was previously proven to improve the symptoms of patients with major depression and with schizophrenia (Huang et al., 2013; Strzelecki et al., 2018). Moreover, sarcosine also ameliorated psychotic symptoms and motor ability in PD patients, thus indicating that it is likely to be involved in the pathogenesis of PD and may therefore represent a promising treatment for PD (Tsai et al., 2014).

Creatine, as a glycine metabolite, has been shown to exert neuroprotective function in neurodegenerative disease. Creatine has been shown to improve motor performance, prevent the loss of DA neurons, and stabilize mitochondrial function in several models of PD (Beal, 2011). In addition, the administration of creatine improves the unified PD rating scale scores of patients (Li et al., 2015). Consistent with these previous data, we detected an elevated creatine level in the CUR-treated group when compared with the MPTP group. The levels of creatine and sarcosine were negatively correlated with the abundance of *Staphylococcaceae*, thus indicating the effect of the gut microbiota on metabolic modulation.

## 5 Conclusion

**Figure 7:**
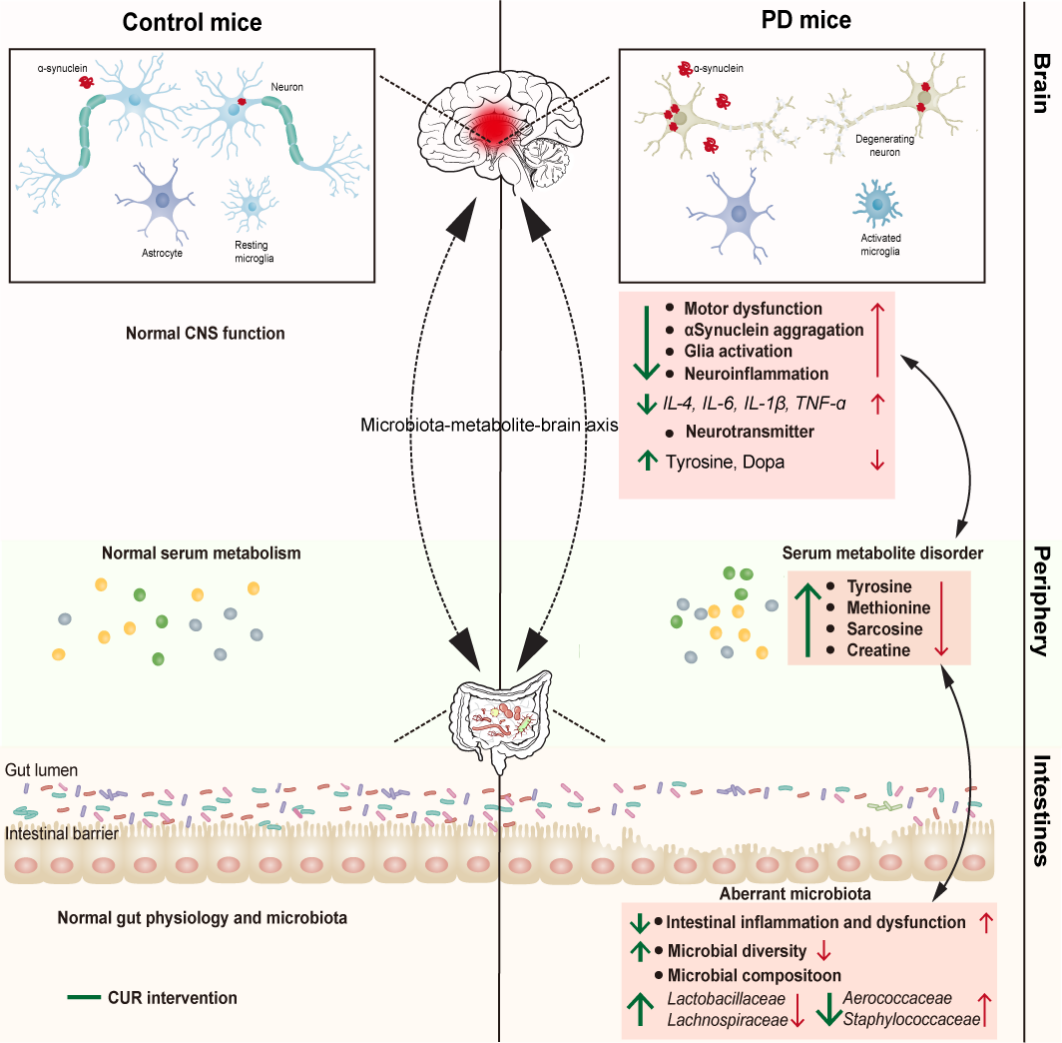
The mechanism by which CUR modulates the gut microbiota-metabolite axis in MPTP-induced PD mouse model. Red arrow represents the changes induced by MPTP treatment, and green arrow indicates the alterations after CUR intervention.

In this study, we demonstrated the preventive effects of CUR on the MPTP-induced model of PD by modulating the gut microbiota community and serum metabolome. Our work highlights an association between the possible CUR-responsive taxa (*Lactobacillaceae*, *Aerococcaceae*) and the tyrosine-dopa axis; these findings could explain, at least in part, the mechanisms underlying the neuroprotective effect of CUR on PD (**Figure 7**).

## Data availability statement

The datasets presented in this study can be found in online repositories. The name of the repository and accession number can be found below: NCBI; PRJNA817373.

## Ethics statement

The animal study was reviewed and approved by Shanghai East Hospital, School of Medicine, Tongji University.

## Author contributions

CC performed the major experiments, analysis and drafted the manuscript, YH accomplished part of the experiments, revision and proof of the manuscript. HL and HY collected the data and performed the analysis. GL, BZ designed the study, supervised the study, double-checked the statistical analysis and revised the manuscript. All authors contributed to the article and approved the submitted version.

## Funding

This work was supported by the Program for Young Excellent Talents in Pudong New Area Health System, Shanghai, China (PWRq2020-10), The Talents Training Program of Shanghai East Hospital (2019xrrcjh06), National Natural Science Foundation of China (grant numbers 82071192, 82101484), Shanghai Science and Technology Innovation Action Plan Project Shanghai Sailing Program (21YF1437600), the Outstanding Leaders Training Program of Pudong New Area Health System, Shanghai, China (PWRL2018-01). The funders had no role in the study design, data collection, data analysis, data interpretation, or writing of the report.

## Acknowledgments

We thank all the patients who participated in this study.

## Conflict of interest

The authors declare that the research was conducted in the absence of any commercial or financial relationships that could be construed as a potential conflict of interest.

## Publisher’s Note

All claims expressed in this article are solely those of the authors and do not necessarily represent those of their affiliated organizations, or those of the publisher, the editors and the reviewers. Any product that may be evaluated in this article, or claim that may be made by its manufacturer, is not guaranteed or endorsed by the publisher.
