# Supplementary material for: Curcumin-driven reprogramming of the gut microbiota and metabolome ameliorates motor deficits and neuroinflammation in a mouse model of Parkinson’s disease
Source: Front Cell Infect Microbiol. 2022 Aug 10;12:887407. doi: 10.3389/fcimb.2022.887407 (PMC9400544; doi:10.3389/fcimb.2022.887407)
Supplement: Supplementary file 1 [file DataSheet_1.zip › Supplemental Figure legends.docx]

**Figure legends**

**Fig. S1 CUR administration suppresses dopaminergic neuronal death and α-synuclein aggregation in MPTP-induced PD mice.** Male C57BL/6 mice were received intragastric administration of CUR (100 mg/kg) once a day for 4 weeks. On day 15, mice were treated with 200 μL saline containing MPTP (30 mg/kg) or 200 μL saline via intraperitoneal injection every day for a total of 5 times to establish PD mice model. On day 28, behavioral tests were performed to evaluate the motor function and the mice were sacrificed to determine the pathology of PD by immunohistochemistry, immunofluorescence and immunoblot. (A, B) Representative images and quantification of Nissl stained cells in the striatum and SNpc among MPTP treated mice, CUR treated mice and controls (n = 5). (C, D) Representative immunohistochemistry images and quantification of pSer129-α-syn-positive cells in the striatum and SNpc (n = 5). Data are expressed as mean ± SEM and representative results are one of the independent experiments. All statistical differences were tested using one-way ANOVA in B, D. Quantification of Nissl-positive cells, pSer129-α-syn-positive cells was performed by ImageJ. **p* < 0.05, ***p* < 0.01, ****p* < 0.001.

**Fig. S2 CUR administration suppresses glial cells activation in the striatum in MPTP-induced mice model.** Male C57BL/6 mice were received intragastric administration of CUR (100 mg/kg) once a day for 4 weeks. On day 15, mice were treated with 200 μL saline containing MPTP (30 mg/kg) or 200 μL saline via intraperitoneal injection every day for a total of 5 times to establish PD mice model. On day 28, the mice were sacrificed to determine the reactive glial cells of PD by immunohistochemistry and immunofluorescence. (A) Upper panel: Representative IF staining of nuclei (DAPI, blue), total dopaminergic neurons (TH, green), and astrocytes (IBA1, red) in the striatum. (A) Representative immunofluorescence images of nuclei (DAPI, blue), dopaminergic neurons (TH, green), and microglia (Iba1, red) in the striatum among MPTP treated mice, CUR treated mice and controls. (B) Quantification of Iba1 positive cells in the striatum using immunofluorescence and immunohistochemistry staining among 3 groups (n = 5). (C) Representative immunofluorescence staining of nuclei (DAPI, blue), dopaminergic neurons (TH, green), and astrocytes (GFAP, red) in the striatum among MPTP treated mice, CUR treated mice and controls. (D) Quantification of GFAP positive cells in the striatum using immunofluorescence and immunohistochemistry staining (n = 5) among 3 groups. (E) The expression of pro-inflammatory genes in the striatum among 3 groups (n = 3-6). Data are expressed as mean ± SEM and representative results are one of the independent experiments. All statistical differences were tested using one-way ANOVA in B, D. Quantification of Iba1-positive cells, GFAP-positive cells was performed by ImageJ. ***p* < 0.01, ****p* < 0.001.

**Fig. 3S CUR administration alters the profile of gut microbiota in the MPTP-induced PD mice model.** Male C57BL/6 mice were received intragastric administration of CUR (100 mg/kg) once a day for 4 weeks. On day 15, mice were treated with 200 μL saline containing MPTP (30 mg/kg) or 200 μL saline via intraperitoneal injection every day for a total of 5 times to establish PD mice model. On day 28, fecal pellets were collected from the mice to detect the gut microbiota by using 16S rRNA sequencing (n = 8-10). (A) Hierarchical clustering tree for each sample at OTU level. (B) Composition of the top 10 bacteria at family level among MPTP treated mice, CUR treated mice and controls by a heatmap. (C) Taxonomic representation of taxa among 3 groups. (D) Histogram of the LDA scores for the microbiota among 3 groups. The threshold of LDA score was 4.0. (E) The scatter points diagrams of COG analysis based on PICRUSt. Data are expressed as mean ± SEM and representative results are one of the independent experiments. All statistical differences were tested using one-way ANOVA in E. **p* < 0.05.

**Fig. S4 Antibiotic pretreatment (ABX) and fecal microbiota transplantation (FMT) validated that the gut microbiota mediated the neuroprotective effect of CUR in the MPTP-induced mice model.** For the ABX treatment experiment, male C57BL/6 mice were received intragastric administration of CUR (100 mg/kg) once a day for 4 weeks starting from day 29. On day 36, mice were treated with 200 μL saline containing MPTP (30 mg/kg) or 200 μL saline via intraperitoneal injection every day for a total of 5 times to establish PD mice model. Mice were received water containing antibiotic solution or regular drinking water alone from the beginning to the end. For the FMT experiment, mice were received water containing antibiotic solution or regular drinking water alone from day1 to day 35. On day 36, FMT administration was performed once a day for 2 weeks.

On day 63, behavioral tests were performed to evaluate the motor function and the mice were sacrificed to determine the pathology of mice by immunofluorescence and immunoblot. (A) Representative immunofluorescence images of TH-positive fibers and neurons in the striatum and SN among ABX MPTP mice, ABX MPTP+CUR mice and ABX controls. (B) Quantification of TH expression in the striatum of ABX MPTP mice, ABX MPTP+CUR mice and ABX controls (n = 3). (C) Quantification of TH-positive fibers and neurons in the striatum and SN among 3 groups. (D) Representative immunofluorescence images of TH-positive fibers and neurons in the striatum and SN among FMT MPTP mice, FMT CUR mice and FMT controls. (E) Quantification of TH expression in the striatum of FMT MPTP mice, FMT CUR mice and FMT controls. (F) Quantification of TH-positive fibers and neurons in the striatum and SN among 3 groups. Data are expressed as mean ± SEM and representative results are one of the independent experiments. All statistical differences were tested using one-way ANOVA in B, C, E, F. Quantification of TH-positive fibers and neurons was performed by ImageJ. **p* < 0.05, ***p* < 0.01, ****p* < 0.001, n.s., not significant.

**Fig. S5 Fecal microbiota transplantation treatment suppresses microglia activation in the SNpc.** For the FMT experiment, mice were received water containing antibiotic solution or regular drinking water alone from day1 to day 35. On day 36, FMT administration was performed once a day for 2 weeks. On day 63, behavioral tests were performed to evaluate the motor function and the mice were sacrificed to determine the pathology of mice by immunofluorescence and immunoblot.

(A) Representative immunofluorescence images of nuclei (DAPI, blue), dopaminergic neurons (TH, green), and microglia (Iba1, red) in the SNpc among FMT MPTP mice, FMT CUR mice and FMT controls. (B) Quantification of Iba1 positive cells in the SNpc using immunofluorescence among 3 groups (n = 3). Data are expressed as mean ± SEM and representative results are one of the independent experiments. All statistical differences were tested using one-way ANOVA in B. **p* < 0.05, ***p* < 0.01.

**Fig. S6 CUR administration attenuated histopathological and functional deterioration in the MPTP-induced mice model.** Male C57BL/6 mice were received intragastric administration of CUR (100 mg/kg) once a day for 4 weeks. On day 15, mice were treated with 200 μL saline containing MPTP (30 mg/kg) or 200 μL saline via intraperitoneal injection every day for a total of 5 times to establish PD mice model. On day 28, the mice were sacrificed to determine the histopathology of the intestine (n = 5-10). (A) Representative H&E staining images of colon, cecum, and rectum among MPTP treated mice, CUR treated mice and controls. (B) Quantification of histological scores of 3 groups. (C) Quantification of the colon length among 3 groups. (D) Total fecal pellets produced in 15 min. (E) Time course of fecal output in a novel environment over 15 min. Data are expressed as mean ± SEM and representative results are one of the independent experiments. All statistical differences were tested using one-way ANOVA in B, C, D, E. **p* < 0.05, ****p* < 0.001.

**Fig. S7 MPTP-induced PD mice manifest different serum metabolites profiles with Control mice.** Male C57BL/6 mice were received intragastric administration of CUR (100 mg/kg) once a day for 4 weeks. On day 15, mice were treated with 200 μL saline containing MPTP (30 mg/kg) or 200 μL saline via intraperitoneal injection every day for a total of 5 times to establish PD mice model. On day 28, serum was collected from the mice to detect the metabolism by using UHPLC-MS (n = 7). (A) The PCoA plot of serum metabonomic between MPTP group and controls. (B) OPLA-DA analysis of the serum metabonomic between MPTP group and controls. (C) The differentially expressed metabolites between MPTP group and controls p using a volcano map. (*VIP* value > 1 and *p* value < 0.05). Each dot represents a detected metabolite. (D) Relative contents of differentially expressed metabolites between MPTP and controls using a heat map. (E) Comparison of KEGG pathway between the MPTP and controls using a bobble plot. Data are expressed as mean ± SEM.
