# Supplementary figures and images for: Curcumin-driven reprogramming of the gut microbiota and metabolome ameliorates motor deficits and neuroinflammation in a mouse model of Parkinson’s disease
Source: Front Cell Infect Microbiol. 2022 Aug 10;12:887407. doi: 10.3389/fcimb.2022.887407 (PMC9400544; doi:10.3389/fcimb.2022.887407)

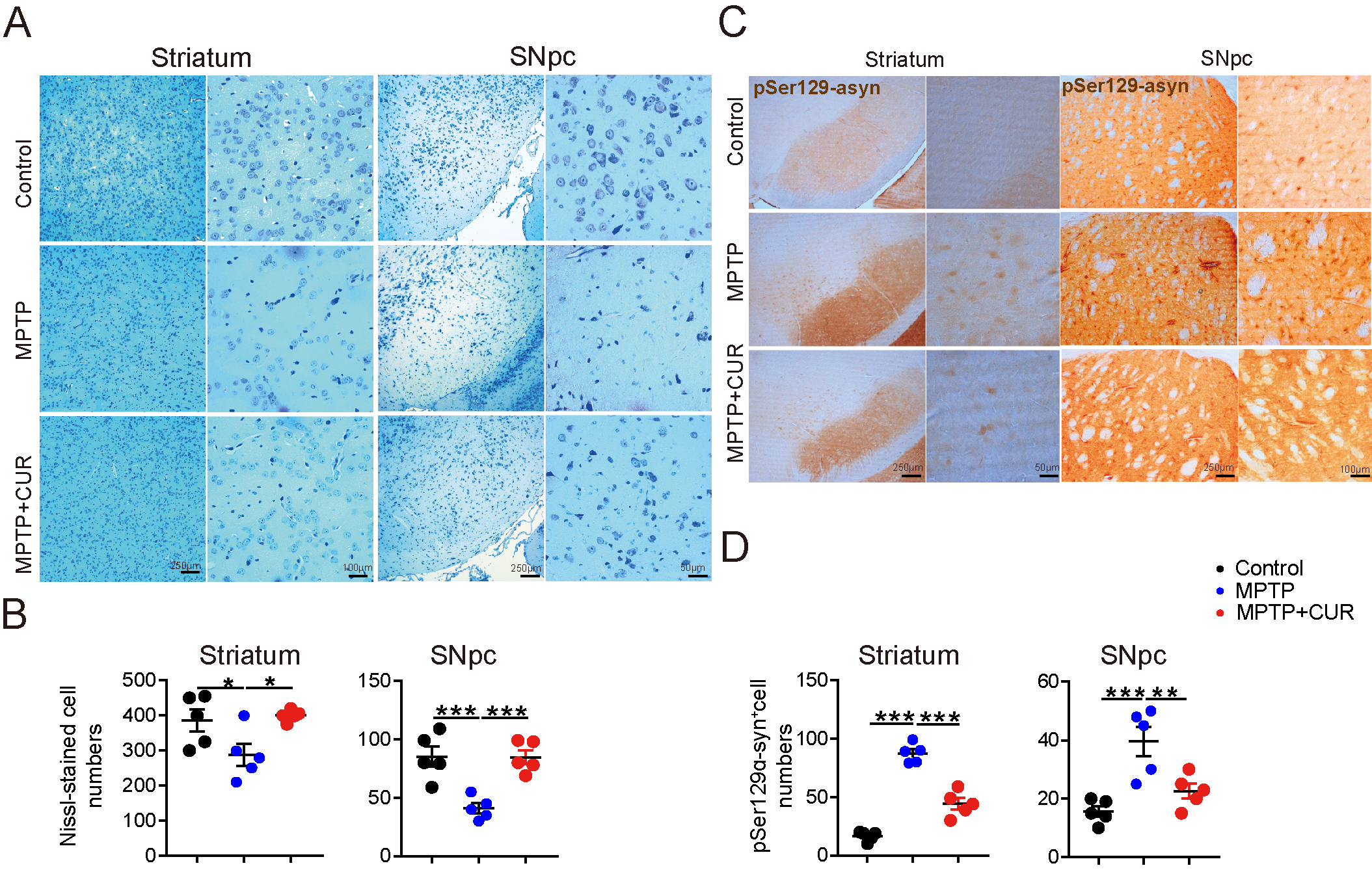

Supplement: Supplementary file 1 [file DataSheet_1.zip › FigureS1.tif]

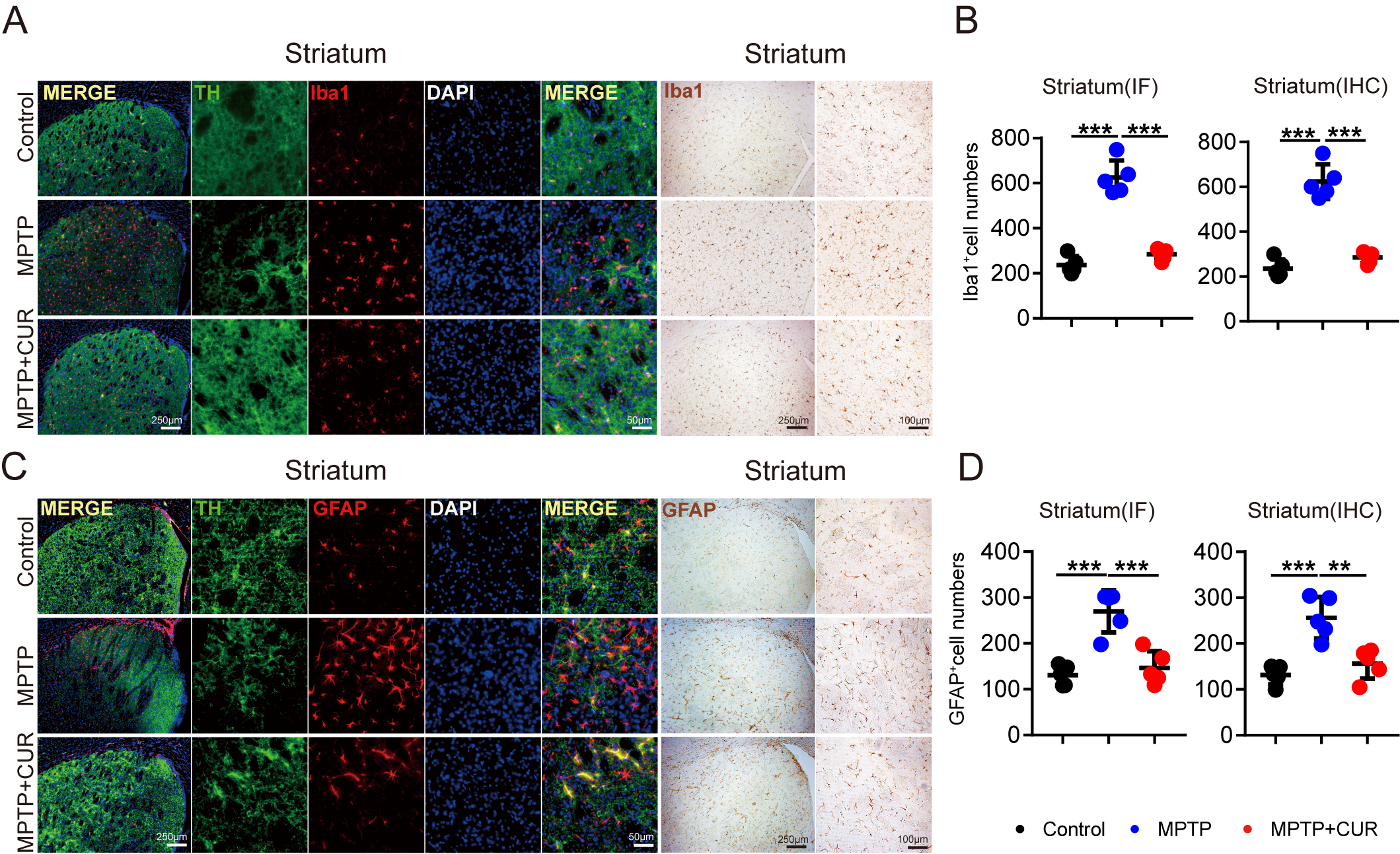

Supplement: Supplementary file 1 [file DataSheet_1.zip › FigureS2.tif]

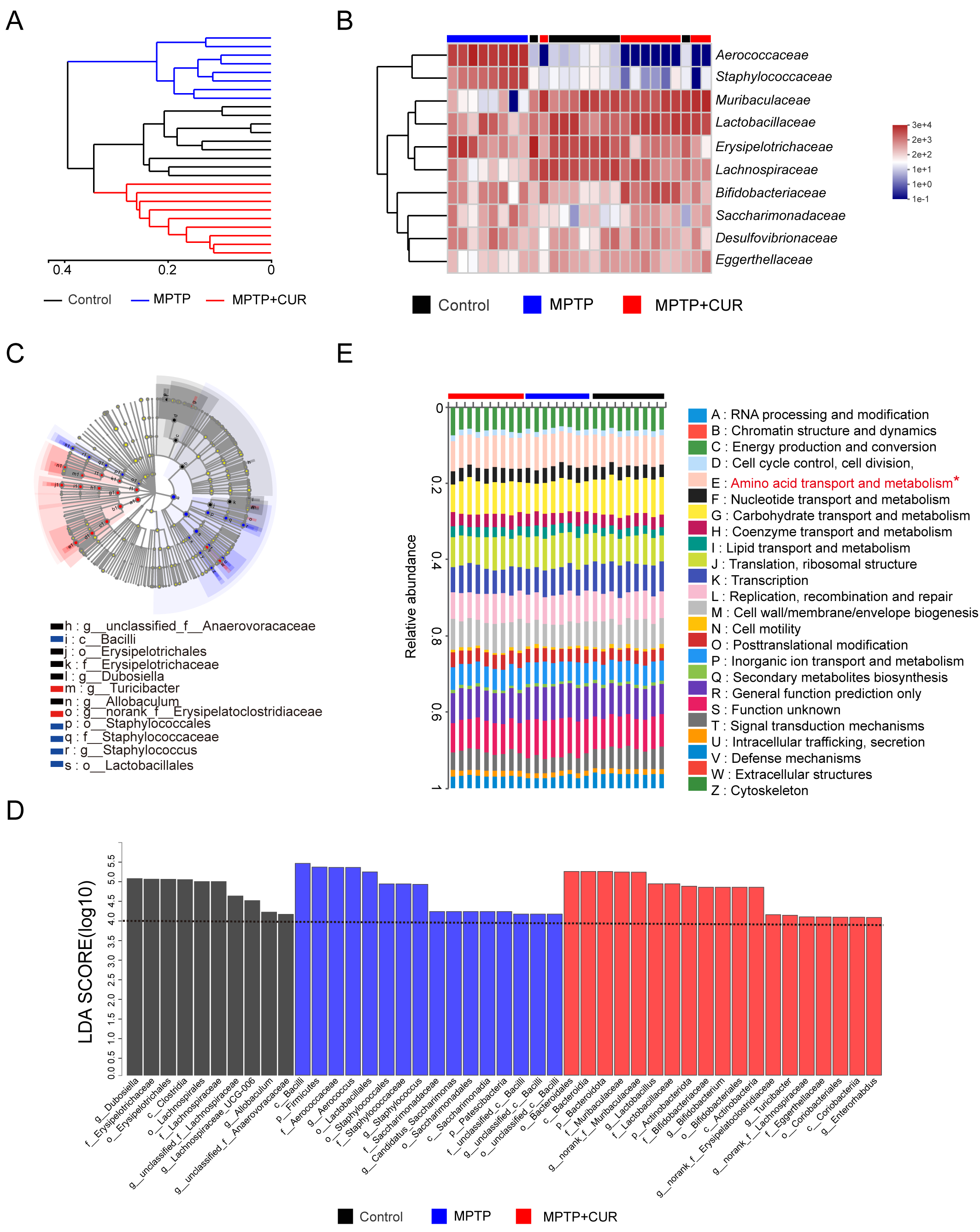

Supplement: Supplementary file 1 [file DataSheet_1.zip › FigureS3.tif]

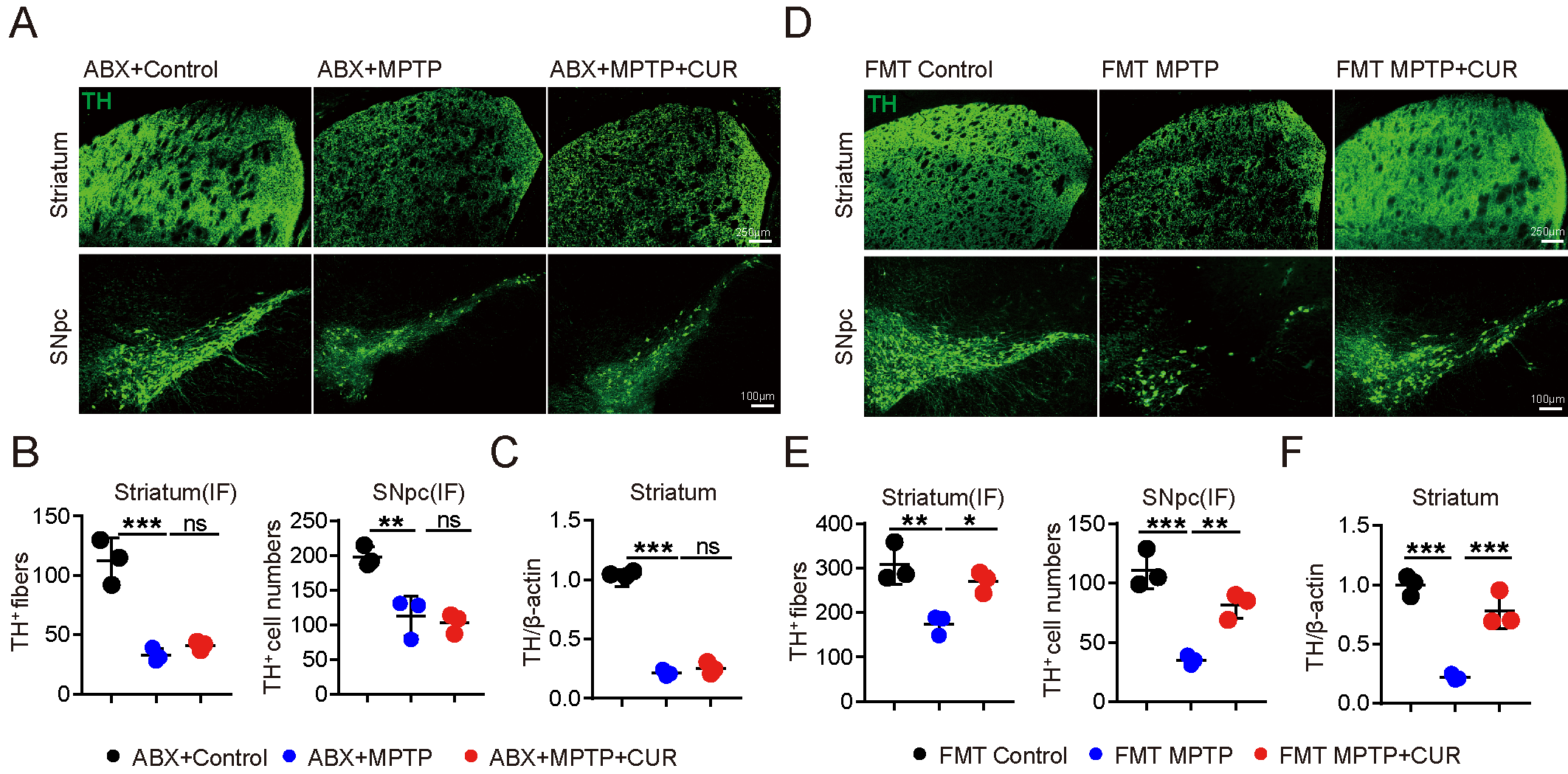

Supplement: Supplementary file 1 [file DataSheet_1.zip › FigureS4.tif]

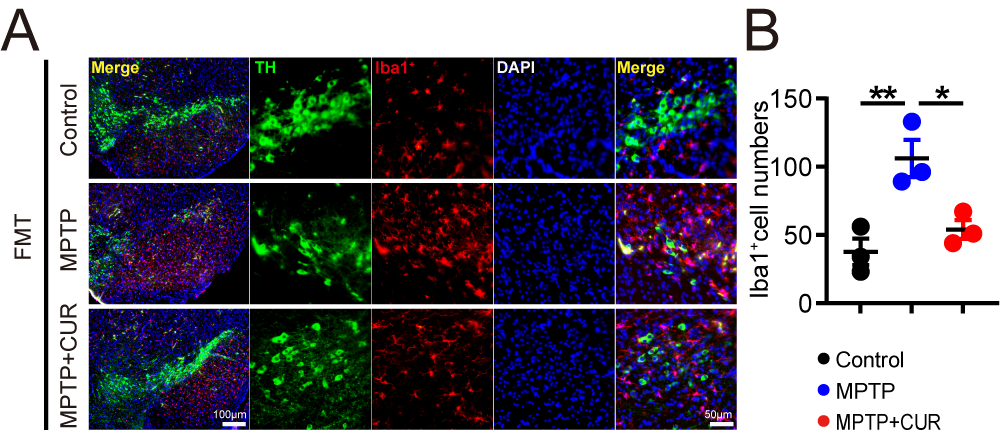

Supplement: Supplementary file 1 [file DataSheet_1.zip › FigureS5.tif]

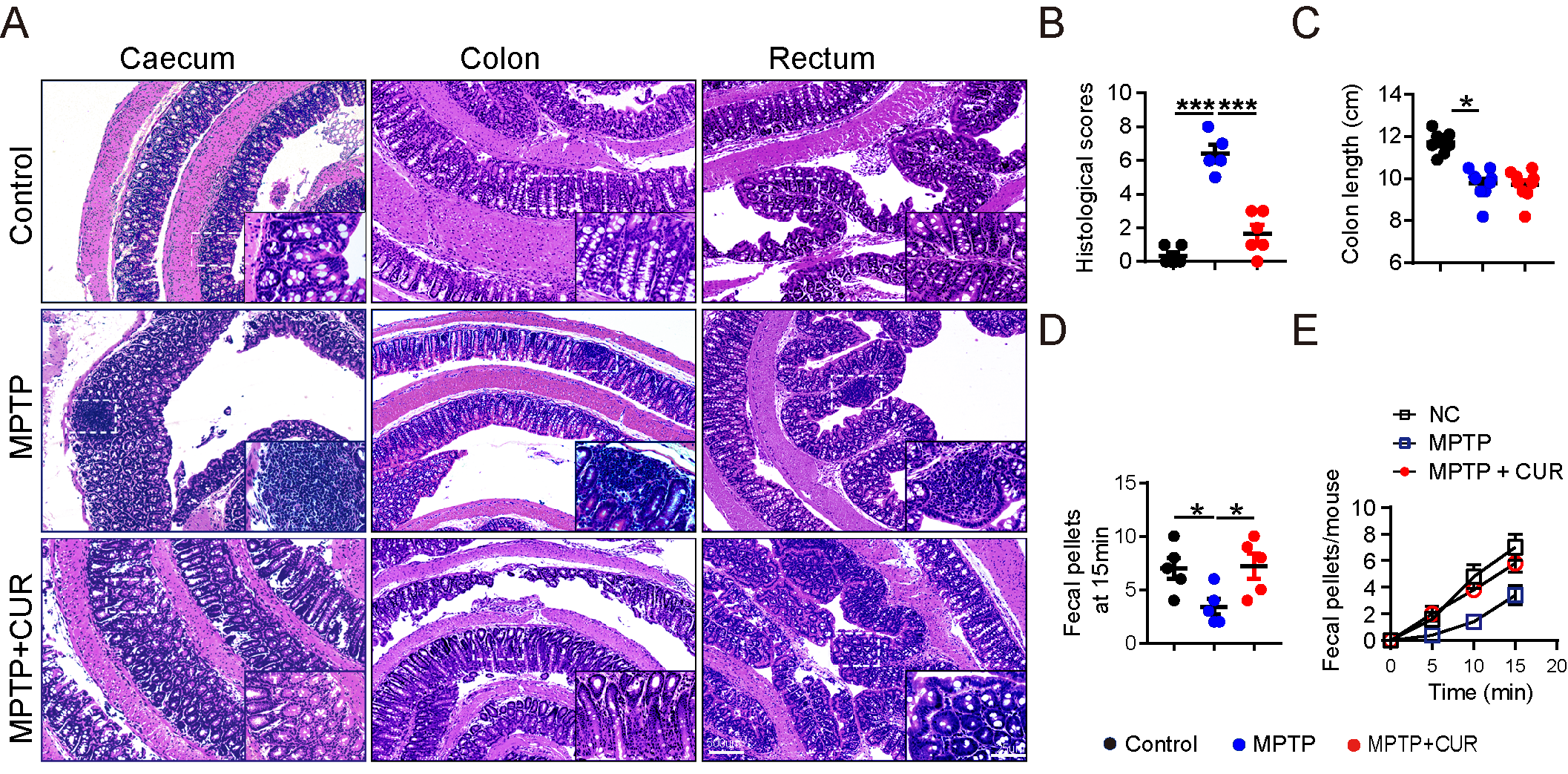

Supplement: Supplementary file 1 [file DataSheet_1.zip › FigureS6.tif]

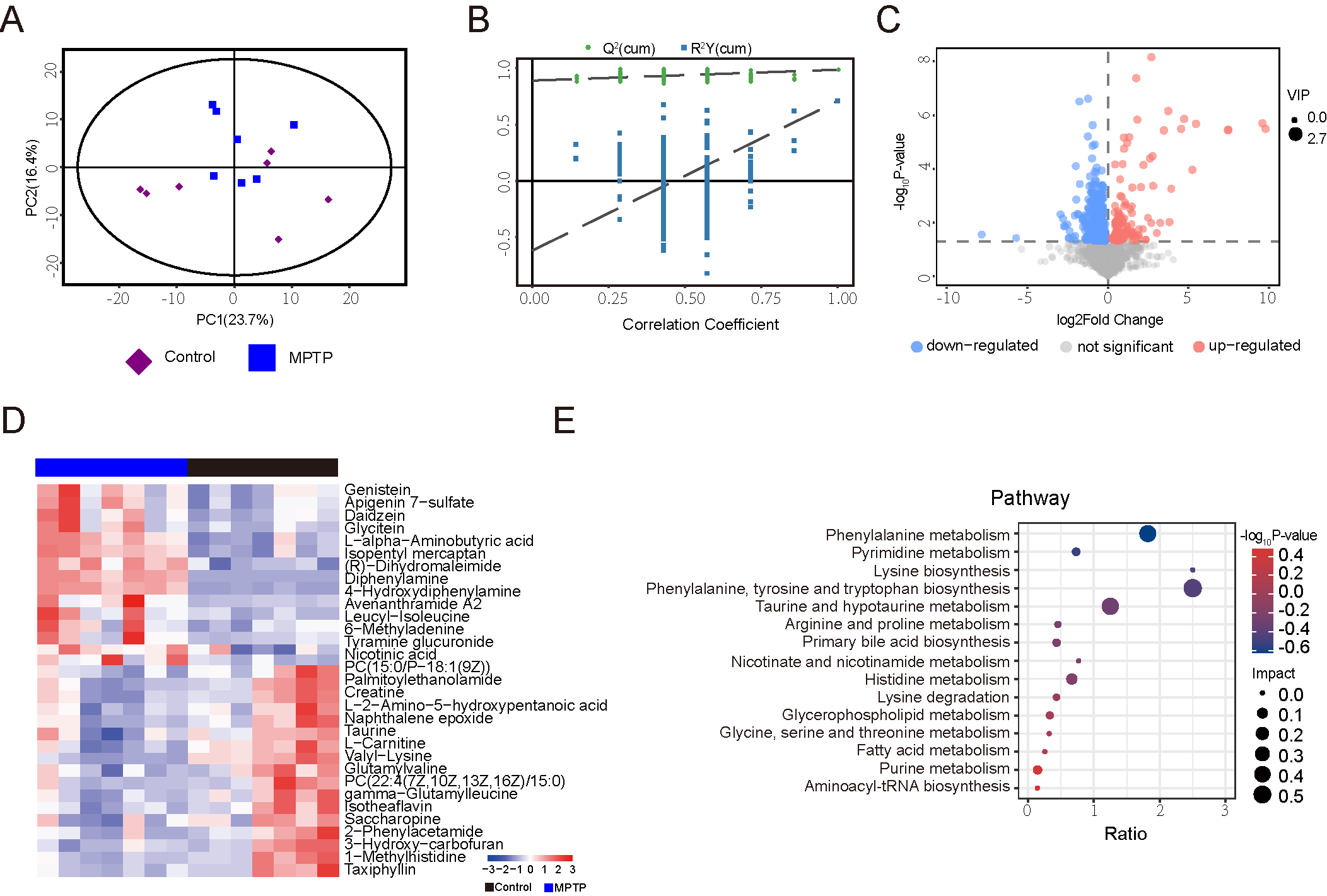

Supplement: Supplementary file 1 [file DataSheet_1.zip › FigureS7.tif]
